# Methylome-wide association study of early life stressors and adult mental health

**DOI:** 10.1093/hmg/ddab274

**Published:** 2021-09-15

**Authors:** David M Howard, Oliver Pain, Ryan Arathimos, Miruna C Barbu, Carmen Amador, Rosie M Walker, Bradley Jermy, Mark J Adams, Ian J Deary, David Porteous, Archie Campbell, Patrick F Sullivan, Kathryn L Evans, Louise Arseneault, Naomi R Wray, Michael Meaney, Andrew M McIntosh, Cathryn M Lewis

**Affiliations:** Social, Genetic and Developmental Psychiatry Centre, Institute of Psychiatry, Psychology & Neuroscience, King's College London, London SE5 8AF, UK; Division of Psychiatry, University of Edinburgh, Royal Edinburgh Hospital, Edinburgh EH10 5HF, UK; Social, Genetic and Developmental Psychiatry Centre, Institute of Psychiatry, Psychology & Neuroscience, King's College London, London SE5 8AF, UK; Social, Genetic and Developmental Psychiatry Centre, Institute of Psychiatry, Psychology & Neuroscience, King's College London, London SE5 8AF, UK; NIHR Maudsley Biomedical Research Centre, South London and Maudsley NHS Trust, London SE5 8AF, UK; Division of Psychiatry, University of Edinburgh, Royal Edinburgh Hospital, Edinburgh EH10 5HF, UK; MRC Human Genetics Unit, Institute of Genetics and Molecular Medicine, University of Edinburgh, Edinburgh EH4 2XU, UK; Centre for Genomic and Experimental Medicine, Institute of Genetics and Cancer, University of Edinburgh, Edinburgh EH4 2XU, UK; Centre for Clinical Brain Sciences, Chancellor’s Building, 49 Little France Crescent, University of Edinburgh, Edinburgh EH16 4SB, UK; Social, Genetic and Developmental Psychiatry Centre, Institute of Psychiatry, Psychology & Neuroscience, King's College London, London SE5 8AF, UK; NIHR Maudsley Biomedical Research Centre, South London and Maudsley NHS Trust, London SE5 8AF, UK; Division of Psychiatry, University of Edinburgh, Royal Edinburgh Hospital, Edinburgh EH10 5HF, UK; Lothian Birth Cohorts, Department of Psychology, University of Edinburgh, Edinburgh EH8 9JZ, UK; Centre for Genomic and Experimental Medicine, Institute of Genetics and Cancer, University of Edinburgh, Edinburgh EH4 2XU, UK; Centre for Genomic and Experimental Medicine, Institute of Genetics and Cancer, University of Edinburgh, Edinburgh EH4 2XU, UK; Usher Institute for Population Health Sciences and Informatics, University of Edinburgh, Edinburgh EH16 4UX, UK; Department of Medical Epidemiology and Biostatistics, Karolinska Institutet, Stockholm 171 77, Sweden; Department of Genetics, University of North Carolina, Chapel Hill, NC 27599, USA; Department of Psychiatry, University of North Carolina, Chapel Hill, NC 27514, USA; Centre for Genomic and Experimental Medicine, Institute of Genetics and Cancer, University of Edinburgh, Edinburgh EH4 2XU, UK; Social, Genetic and Developmental Psychiatry Centre, Institute of Psychiatry, Psychology & Neuroscience, King's College London, London SE5 8AF, UK; Queensland Brain Institute, University of Queensland, Brisbane, Queensland 4072, Australia; Douglas Hospital Research Centre, Douglas Mental Health University Institute, McGill University, Montréal, QC H4H 1R3, Canada; Ludmer Centre for Neuroinformatics and Mental Health, McGill University, Montréal, QC H3T 1E2, Canada; Singapore Institute for Clinical Sciences, Singapore 117609, Singapore; Division of Psychiatry, University of Edinburgh, Royal Edinburgh Hospital, Edinburgh EH10 5HF, UK; Lothian Birth Cohorts, Department of Psychology, University of Edinburgh, Edinburgh EH8 9JZ, UK; Social, Genetic and Developmental Psychiatry Centre, Institute of Psychiatry, Psychology & Neuroscience, King's College London, London SE5 8AF, UK; NIHR Maudsley Biomedical Research Centre, South London and Maudsley NHS Trust, London SE5 8AF, UK

## Abstract

The environment and events that we are exposed to in utero, during birth and in early childhood influence our future physical and mental health. The underlying mechanisms that lead to these outcomes are unclear, but long-term changes in epigenetic marks, such as DNA methylation, could act as a mediating factor or biomarker. DNA methylation data were assayed at 713 522 CpG sites from 9537 participants of the Generation Scotland: Scottish Family Health Study, a family-based cohort with extensive genetic, medical, family history and lifestyle information. Methylome-wide association studies of eight early life environment phenotypes and two adult mental health phenotypes (major depressive disorder and brief resilience scale) were conducted using DNA methylation data collected from adult whole blood samples. Two genes involved with different developmental pathways (*PRICKLE2*, Prickle Planar Cell Polarity Protein 2 and *ABI1*, Abl-Interactor-1) were annotated to CpG sites associated with preterm birth (*P* < 1.27 × 10^−9^). A further two genes important to the development of sensory pathways (*SOBP*, Sine Oculis Binding Protein Homolog and *RPGRIP1*, Retinitis Pigmentosa GTPase Regulator Interacting Protein) were annotated to sites associated with low birth weight (*P* < 4.35 × 10^−8^). The examination of methylation profile scores and genes and gene-sets annotated from associated CpGs sites found no evidence of overlap between the early life environment and mental health conditions. Birth date was associated with a significant difference in estimated lymphocyte and neutrophil counts. Previous studies have shown that early life environments influence the risk of developing mental health disorders later in life; however, this study found no evidence that this is mediated by stable changes to the methylome detectable in peripheral blood.

## Introduction

The diathesis-stress model posits that behaviours and psychological disorders are the result of underlying biological factors (diatheses) plus exposure to stressful events or environments. Childhood adversity increases the risk of poorer physical and mental health outcomes in later life (1,2), with neglect, sexual and emotional abuse, and violence providing greater risk (3–6). However, multiple studies have reported additional perinatal and early life stressors and environments that are also associated with adult mental health. Individuals born preterm or with a low birth weight are more likely to experience problems with attentiveness and hyperactivity, as well as elevated levels of anxiety and depression (7–9). Seasonality of birth has also been associated with psychiatric outcomes in later life (10–12), with those born in January having higher risk of schizophrenia and bipolar and those born in June and July having greater risk of depression. Parental factors, including the age of the parents at birth (13) or having an absent parent (14), are reported to increase the risk of depression. Where we live has also been shown to be detrimental to mental health (15) and is likely due to a variety of environmental and social factors (16,17).

Adverse environments in the gestational and postnatal periods are known to cause long-term alterations to DNA methylation across the genome (18). These modifications are reported to mediate resilience and responses to stress-related disorders throughout the life course (19). Epigenomic variation has also been implicated in a range of psychiatric conditions (20), including bipolar disorder (21), schizophrenia (22) and major depressive disorder [MDD (23)]. Much of the epigenetic research seeking to link early life environments and MDD has been conducted using small cohort samples sizes (typically <1000) and focussed on candidate regions (24). However, this approach is likely to be suboptimal as demonstrated by the lack of reproducible results from candidate gene association studies for MDD (25).

The principal aim of the current research was to investigate whether alterations to DNA methylation have the potential to mediate the stress component in the diathesis-stress model. This was achieved by conducting a methylome-wide association study (MWAS) of early life environments and later mental health outcomes in a single study cohort of over 9500 adults from the Generation Scotland: Scottish Family Health Study cohort (26). Linked electronic health records, responses at interview and questionnaire data were used to ascertain the early life environments, MDD status and psychological resilience measured using the brief resilience scale (BRS). MDD and BRS were examined due to their reported association with early life environments (27,28). The associated CpG sites identified by the MWAS of these phenotypes were then annotated to genes and gene-sets. The gene and gene-set overlap between the early life environments and mental health outcomes were then examined. In addition, methylation profile scores were used to assess the broader methylome-wide overlap between the early life environment and mental health traits.

## Results

We conducted analyses of eight early life environments (preterm birth, low birth weight, birth month, birth date, having a young parent, having a lone parent, urban environment, and population density) and two measures of adult mental health (MDD and BRS) using the Generation Scotland: Scottish Family Health Study cohort (*n* = 24 080). First, we used regression to assess the association between the early life environments and MDD and BRS using phenotypic data. Second, we conducted a MWAS to identify CpG sites associated with each phenotype. Finally, we used the summary statistics from the MWAS to determine the extent of any overlap between the early life environments and the mental health phenotypes using genes and gene-set analysis and methylation profile scores.

### Regression of adult mental health on early life environments

Generalized linear mixed models were used to assess each early life environment in turn and its association with either MDD or BRS (Supplementary Material, Table 1), after adjusting for sex and relatedness between individuals. There were no significant associations between the early life environments and the adult mental health phenotypes after adjusting for multiple testing (*P* > 6.25 × 10^−3^). All early life environments, except for preterm birth, marginally increased the risk of developing MDD in adulthood. All early life environments, except for having a lone parent, marginally lowered BRS suggesting a negative effect on psychological resilience. The associations between birth date and birth month with BRS were nominally significant (*P* < 0.05) with those born across the summer months scoring lower on the BRS; however, these associations did not remain after correction for multiple testing.

### MWAS of early life environments and adult mental health

To identify CpG sites associated with each of the early life environments and the mental health phenotypes a MWAS was conducted. Normalized *M*-values for 713 522 CpG sites profiled from combined blood samples of 9537 GS:SFHS individuals (5087 in Set 1 and 4450 in Set 2) remained after quality control procedures. The associations between these *M*-values and the early life environments and mental health phenotypes were estimated using two association study methods (MWAS 1 and MWAS 2). MWAS 1 fits a linear regression model to the data with the *M*-values as the dependent variable, whereas MWAS 2 fits the phenotype as the dependent variable while accounting for methylome-wide correlational structure and with an additional correction for predicted blood cell type composition.

The significant CpG sites (*P* < 7.01 × 10^−8^) from MWAS 1 and MWAS 2 are in Table 1, except those associated with birth month and with birth date which are in Supplementary Material, Tables 2 and 3. In Table 1, the six significant CpG sites identified in MWAS 2 were also significant in MWAS 1 for the same phenotypes. In MWAS 1, there were 93 significant sites for birth month and 637 significant sites for birth date. However, no CpG sites were significant in MWAS 2 for either birth month or birth date; further analysis examining this discrepancy is covered in the Supplementary Information.

**Table 1:** CpG sites associated with phenotypes in either MWAS 1 or MWAS 2

Phenotype	CpG site	Chr	BP position	Annotated Gene	MWAS 1			MWAS 2		
					Effect size	Standard error	*P*-value	Effect size	Standard error	*P*-value
Preterm birth	cg00725333	3	64 189 256	*PRICKLE2*	−0.2747	0.0376	**6.24 × 10** ^**−13**^	−0.0364	0.0060	**1.15 × 10** ^**−9**^
	cg17668848	3	108 029 973	*HHLA2*	−0.2871	0.0396	**9.13 × 10** ^**−13**^	−0.0356	0.0058	**8.21 × 10** ^**−10**^
	cg24329141	10	27 095 369	*ABI1*	−0.2368	0.0352	**2.98 × 10** ^**−11**^	−0.0345	0.0057	**1.27 × 10** ^**−9**^
Low birth weight	cg15582176	1	1 183 528	*C1QTNF12*	−0.3604	0.0661	**6.53 × 10** ^**−8**^	−0.0254	0.0054	2.49 × 10^−6^
	cg19909717	6	107 924 415	*SOBP*	−0.2225	0.0403	**4.35 × 10** ^**−8**^	−0.0329	0.0055	**1.59 × 10** ^**−9**^
	cg21803443	7	126 547 786	*GRM8*	−0.3656	0.0618	**4.62 × 10** ^**−9**^	−0.0278	0.0056	7.22 × 10^−7^
	cg12090821	14	21 755 658	*RPGRIP1*	−0.0857	0.0152	**2.43 × 10** ^**−8**^	−0.0329	0.0058	**1.47 × 10** ^**−8**^
	cg05905731	16	85 485 785		−0.1695	0.0300	**2.23 × 10** ^**−8**^	−0.0294	0.0055	7.50 × 10^−8^
Young parent	cg00528572	11	92 703 433	*MTNR1B*	−0.1369	0.0200	**9.04 × 10** ^**−12**^	−0.0145	0.0025	**4.47 × 10** ^**−9**^
	cg02427109	18	77 917 459	*PARD6G, PARD6G-AS1*	−0.1163	0.0213	**4.96 × 10** ^**−8**^	−0.0101	0.0025	4.12 × 10^−5^
Population density	cg06759845	1	156 460 474	*MEF2D*	5.20 × 10^−6^	9.40 × 10^−7^	**3.40 × 10** ^**−8**^	90.39	17.02	1.10 × 10^−7^
	cg12433043	6	100 619 893		−6.65 × 10^−6^	1.20 × 10^−6^	**3.37 × 10** ^**−8**^	−88.86	16.75	1.13 × 10^−7^
	cg03623878	13	113 655 560	*MCF2L*	9.10 × 10^−6^	1.58 × 10^−6^	**9.56 × 10** ^**−9**^	81.18	16.88	1.51 × 10^−6^
	cg08036492	17	13 976 536	*COX10*	3.09 × 10^−6^	5.71 × 10^−7^	**6.74 × 10** ^**−8**^	87.75	18.29	1.61 × 10^−6^
MDD	cg02280719	1	6 802 222		0.0400	0.0064	**5.99 × 10** ^**−10**^	0.0174	0.0043	5.32 × 10^−5^
	cg08548783	6	57 903 690		0.0378	0.0067	**1.37 × 10** ^**−8**^	0.0142	0.0043	9.79 × 10^−4^

*Note:* MWAS 1 results are from a methylome-wide association analysis using linear regression and MWAS 2 results are from a methylome-wide association analysis using OSCA. Bold *P*-values indicate an association after correction for multiple testing (*P* < 7.01 × 10^−8^). Sites are ordered by phenotype and then genomic position. Chromosome number (Chr) and base pair (BP) position are based on genome assembly GRCh37 (hg19). Annotation of genes is provided by missMethyl.

Miami plots for preterm birth, low birth weight, having a young parent, population density, and MDD are in Figures 1–5, respectively. The remaining Miami plots are in Supplementary Material, Figures 1–5 and QQ-plots and $\lambda$ for all phenotypes are in Supplementary Material, Figures 6–15.

**Figure 1:**
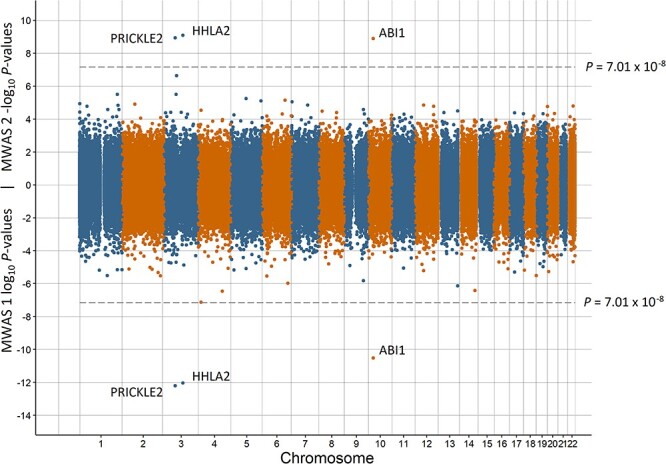
Miami plot of the observed *P*-values of each CpG site for an association with preterm birth. Log_10_*P*-values are shown for MWAS 1 and −log_10_*P*-values are shown for MWAS 2. The dotted lines indicate methylome-wide significance (*P* = 7.01 × 10^−8^). The annotation of genes for significant sites is reported by missMethyl for MWAS 1 and by OSCA for MWAS 2.

**Figure 2:**
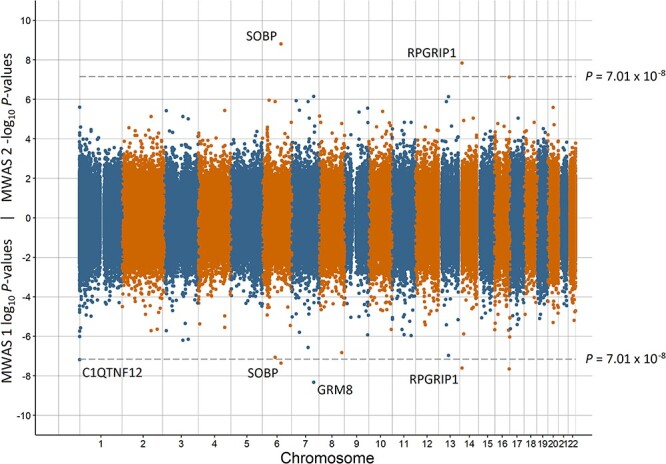
Miami plot of the observed *P*-values of each CpG site for an association with low birth weight. Log_10_*P*-values are shown for MWAS 1 and −log_10_*P*-values are shown for MWAS 2. The dotted lines indicate methylome-wide significance (*P* = 7.01 × 10^−8^). The annotation of genes for significant sites is reported by missMethyl for MWAS 1 and by OSCA for MWAS 2.

**Figure 3:**
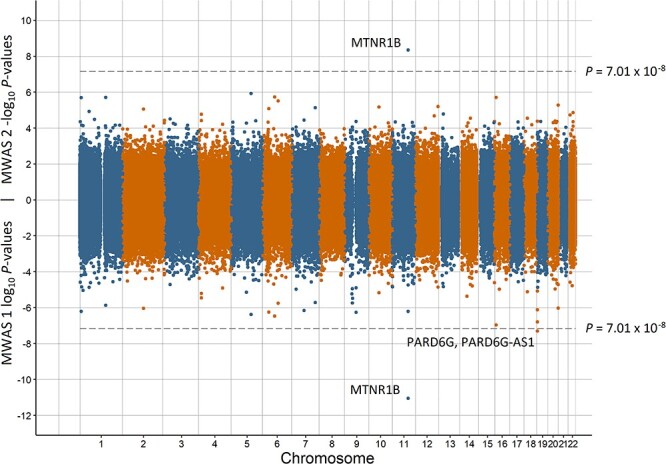
Miami plot of the observed *P*-values of each CpG site for an association with having a young parent. Log_10_*P*-values are shown for MWAS 1 and −log_10_*P*-values are shown for MWAS 2. The dotted lines indicate methylome-wide significance (*P* = 7.01 × 10^−8^). The annotation of genes for significant sites is reported by missMethyl for MWAS 1 and by OSCA for MWAS 2.

**Figure 4:**
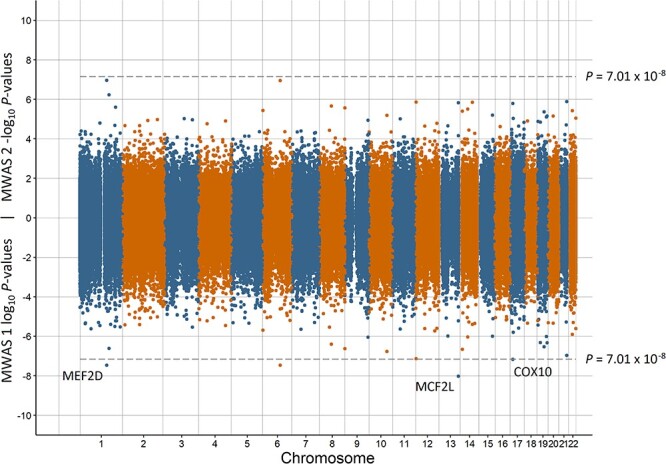
Miami plot of the observed *P*-values of each CpG site for an association with population density. Log10 *P*-values are shown for MWAS 1 and −log10 *P*-values are shown for MWAS 2. The dotted lines indicate methylome-wide significance (*P* = 7.01 × 10^−8^). The annotation of genes for significant sites is reported by missMethyl for MWAS 1.

**Figure 5:**
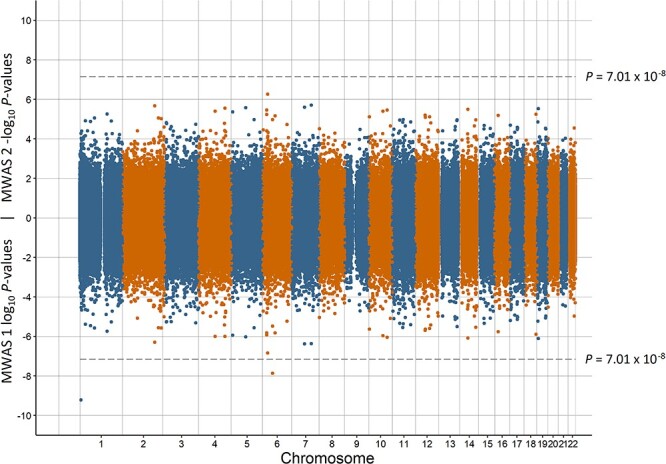
Miami plot of the observed *P*-values of each CpG site for an association with MDD. Log_10_*P*-values are shown for MWAS 1 and −log_10_*P*-values are shown for MWAS 2. The dotted lines indicate methylome-wide significance (*P* = 7.01 × 10^−8^). Significant sites did not annotate to any genes.

### Gene and gene-set analysis

The genes annotated to associated CpG sites from MWAS 1 and MWAS 2 were examined for overlap between the early life environment and adult mental health phenotypes. For the significant sites in MWAS 1, there were three annotated genes for preterm birth, having a young parent and population density, four annotated genes for low birth weight, 20 annotated genes for birth month and 167 annotated genes for birth date. There were no annotated genes for the mental health phenotypes and therefore no overlap was observed with the early life environment phenotypes.

For significant sites in MWAS 2, there were three annotated genes for preterm birth, two annotated genes for low birth weight and one annotated gene for having a young parent. As there were no annotated genes for the mental health phenotypes, there was no overlap of annotated genes with the early life environment phenotypes.

There were no enriched Gene Ontology or the KEGG gene-sets (*P* > 0.05) for any of the phenotypes from either MWAS 1 or MWAS 2.

### Mediation analysis

cg21803443 was associated with low birth weight in MWAS 1 and was annotated to the *GRM8* (Glutamate Metabotropic Receptor 8) protein-coding gene. Küpers *et al.* (29) also reported a CpG site (cg15908975) annotated to *GRM8* associated with birth weight. *GRM8* has also been associated with depression (30). Therefore, a mediation analysis with low birth weight as the independent variable, either cg21803443 or cg15908975 as the mediator variable, and MDD as the dependent variable was conducted. There were no significant direct or indirect effects (*P* > 0.05; Supplementary Material, Figs 16 and 17) when fitting either CpG site as the mediator variable.

### Methylation profile scores

The MWAS 2 effect size estimates from Set 1 results for the early life environment phenotypes were used to calculate profile scores for individuals in Set 2. The utility of these profile scores for predicting MDD, BRS or the same phenotype (Table 2) in Set 2 was assessed. There was significant prediction of birth month using birth month profile scores (*R*^2^ = 0.002, *P* = 5.19 × 10^−3^) and of having a young parent using the profile scores from young parent (*R*^2^ = 0.013, *P* = 3.46 × 10^−5^). There was nominal prediction of MDD from preterm birth (*R*^2^ = 0.002, *P* = 0.034) and birth month (*R*^2^ = 0.002, *P* = 0.043), and nominal prediction of BRS from birth month (*R*^2^ = 0.003, *P* = 0.016). However, none of the profile scores predicting mental health phenotypes were significant after correction for multiple testing (*P* > 6.25 × 10^−3^). The MWAS results from a continuous measure of gestation length conducted by Merid *et al.* (31) and from a continuous measure of birth weight conducted by Küpers *et al.* (29) were used to construct profile scores in GS:SFHS; however, these score was not associated with either MDD or BRS (*P* > 0.05).

**Table 2:** Prediction of MDD, brief resilience scale and the same early life environment in Set 2 using methylation profile scores

	MDD			Brief resilience scale			Same early life environment		
Early life environment	Threshold	*R* ^2^	*P*-value	Threshold	*R* ^2^	*P*-value	Threshold	*R* ^2^	*P*-value
Preterm birth	1 × 10^−4^	0.0023	0.034	1 × 10^−7^	0.0001	0.74	1 × 10^−7^	0.0575	0.11
Low birth weight	1 × 10^−5^	0.0005	0.32	1 × 10^−6^	0.0009	0.21	1 × 10^−7^	0.0177	0.26
Birth month	1 × 10^−6^	0.0021	0.043	1 × 10^−6^	0.0032	0.016	1 × 10^−4^	0.0024	**5.19 × 10** ^**−3**^
Birth date	1 × 10^−5^	0.0013	0.10	1 × 10^−5^	0.0016	0.09	1 × 10^−4^	0.0009	0.042
Young parent	1 × 10^−3^	0.0006	0.28	1 × 10^−5^	0.0010	0.19	1 × 10^−5^	0.0128	**3.46 × 10** ^**−5**^
Lone parent	1 × 10^−5^	0.0005	0.34	1 × 10^−4^	0.0001	0.66	1 × 10^−3^	0.0004	0.49
Urban environment	1 × 10^−5^	0.0004	0.40	1 × 10^−2^	0.0008	0.25	1 × 10^−5^	0.0006	0.21
Population density	1 × 10^−4^	0.0015	0.08	1 × 10^−6^	0.0010	0.19	1 × 10^−5^	0.0013	0.037

*Notes:* Scores were calculated using the CpG site effect sizes from MWAS 2 of early life environment phenotypes in Set 1. The effect sizes in Set 1 were obtained from methylome-wide association studies using OSCA (MWAS 2). Multiple thresholds based on CpG site *P*-values were examined, with the threshold explaining the greatest phenotypic variance reported. An adjusted *R*^2^ from a simple linear regression was used for the prediction of quantitative phenotypes, else Nagelkerke’s *R*^2^ was used to calculate the proportion of phenotypic variance explained on the liability scale. Bold *P*-values indicate an association after correction for multiple testing (*P* < 6.25 × 10^−3^).

## Discussion

The impact of early life environments on later life is of critical importance across multiple clinical and research domains. We sought to examine eight early life environments experienced around the time of birth and quantify: (i) their association with mental health in adulthood; (ii) whether they were associated with detectable changes to the methylome in to adulthood; and (iii) whether there was any shared association on the methylome between early life environments and adult mental health.

Previous studies have reported an effect of extreme preterm birth (gestation length <28 weeks) on mental health (8,32). The population-based GS:SFHS cohort had four participants (out of 3134) born at <28 weeks gestation length and therefore a 37-week threshold for a preterm birth was used. Three CpG sites were associated with preterm birth in both the MWAS analyses: cg00725333, cg17668848 and cg24329141. These three sites mapped to *PRICKLE2* (Prickle Planar Cell Polarity Protein 2), *HHLA2* (Human Endogenous Retrovirus-H Long Terminal Repeat-Associating Protein 2) and *ABI1* (Abl-Interactor-1) protein coding genes, respectively. In humans, *PRICKLE2* has been associated with myelomeningocele (33), a severe form of spina bifida. Myelomeningocele compromises the development of the spine and spinal cord in the womb and has been associated with preterm birth (34). *HHLA2* regulates T-cell function (35), however its role in gestation length is unclear. Knockouts of *ABI1* in mice have demonstrated it to be essential for embryonic development, survival (36) and placental development (37). *PRICKLE2* and *ABI1* have plausible roles in gestation length with altered methylation of proximal CpG sites into adulthood. A MWAS conducted using preterm infants (38) revealed a different set of associated CpG sites to those observed here in adulthood.

Similar to preterm birth, published studies of birth weight and mental health report that lower birth weight thresholds identify stronger associations with poorer mental health (39,40). In the current study, a sex and gestational-age adjusted measure of birth weight was used increasing the distinction with the preterm birth phenotype. Five CpG sites were associated with birth weight in MWAS 1 of which two were also significant in MWAS 2. The two sites that were significant in both MWAS annotated to genes important for the development of the sensory systems: *SOBP* (Sine Oculis Binding Protein Homolog) and *RPGRIP1* (Retinitis Pigmentosa GTPase Regulator Interacting Protein). *SOBP* has been implicated in the embryonic development of the mouse cochlea (41). In a meta-analysis of DNA methylation data in neonates for low birth weight, a CpG site annotated to the *SOBP* gene was also significant after correcting for false discovery rate [but not after Bonferroni correction; (29)]. The critical role of *RPGRIP1* in the remodelling of rod photoreceptors has been demonstrated in humans (42). The association between low birth weight and the long-term expression of CpG sites requires further investigation, certainly as low birth weight has been associated with both hearing impairment (43) and ophthalmic deficits (44). cg21803443 on chromosome 7 was significant in MWAS 1 and annotated to the *GRM8* protein-coding gene. Küpers *et al.* (29) also identified a CpG site (cg15908975, *P* = 4.52 × 10^−7^ in a European meta-analysis) close to *GRM8* associated with birth weight. *GRM8* is involved in the inhibition of the cyclic AMP cascade influencing glutamatergic neurotransmission and had an association with depression (*P* = 1.80 × 10^−12^) in a genome-wide association meta-analysis (30). Mediation analysis of two CpG sites close to GRM8 (cg21803443 and cg15908975), fitting birth weight as the independent variable and MDD as the dependent variable did not reveal a significant direct or indirect effect. There was no predictive ability of a methylation profile score for low birth weight to predict either MDD or BRS. A previous analysis of birth weight as a continuous trait in GS:SFHS identified one significant CpG site [cg00966482; (45)], however this site was not significant in either MWAS 1 (*P* = 0.22) or MWAS 2 (*P* = 0.53).

Schnittker (12) suggests that the role of seasonality of birth on mental health was more prevalent in the early part of the 20th century and was partly attributable to poorer prenatal nutrition across the winter months. However, Disanto *et al.* (10) analysed post-1950 data from England and reported an effect of seasonality of birth on schizophrenia, bipolar disorder and to a lesser extent recurrent MDD. In the current study, there was nominal evidence for an effect of birth date and birth month on BRS and nominal prediction of both MDD and BRS using a profile score for birth month calculated from DNA methylation data. The greatest difference between the MWAS 1 and MWAS 2 results was for birth date and birth month. Additional prediction into the Lothian Birth Cohorts of 1921 and 1936 [LBC (46); see Supplementary Information], using methylation profile scores, demonstrated that this divergence is likely due to differing blood cell type composition (for neutrophils and lymphocytes) between those born in the winter and those born in the summer. Altered gene expression due to seasonality has been reported previously (47), with seasonality of birth also influencing neonatal immune development (48) and thymic output (49). The current work provides further substantial evidence that birth date is associated with blood cell type composition. This association is detectable throughout the life course and needs to be accounted for in future research on traits influenced by seasonality of birth.

In the present study, there was no effect of having a young parent on mental health. However, a much larger study (2.9 M individuals) on Danish participants found an increased risk of mood disorders (International Classification of Disease codes F30–39) for those with teenage mothers [incidence risk ratio = 1.35 (95% CI = 1.30–1.40)] and those with teenage fathers [incidence risk ratio = 1.20 (95% CI = 1.13–1.27); (13)]. The methylation profile for having a young parent from Set 1 had significant prediction of having a young parent in Set 2, suggesting replication across Sets.

Two correlated early life phenotypes, urban environment and population density, were studied to measure the effect of geographical environment at birth. There was no effect of either early life phenotype on MDD or BRS and there were no associated CpG sites. Urbanicity has been associated with depression (50,51), although no association has been observed in low- and middle-income countries (52) and the United States (53). The contradictory findings and the lack of observable effects here may be due to the multiple factors incorporated in these phenotypes, such as pollution (54) and socio-economic status (55), but may be offset by access to mental health services.

Published DNA methylation analyses of MDD have typically been conducted on relatively small samples. The two CpG sites associated with MDD in the current study did not annotate to known protein coding genes. These two sites were outside of the ten differentially methylated regions for major depression identified by Roberson-Nay *et al.* (56) (39 major depression cases and 111 controls) and located away from the locations identified for MDD by Oh *et al.* (57) (103 cases and 97 controls) and Starnawska *et al.* (58) (724 individuals assessed for depression symptomatology score). A longitudinal MWAS of MDD (199 cases and 382 in remittance) identified six plausible CpG sites based on function (59), although none of these sites remained significant after applying correction for multiple testing. A further study of profile scores for MDD in GS:SFHS found that prediction of MDD in an independent subset was possible, but was reliant on capturing lifestyle factors associated with MDD (60). An association meta-analysis (7948 individuals) identified 20 CpG sites with a suggestive association (*P* < 1 × 10^−5^) with depressive symptoms (23), of which one was nominally significant (*P* = 0.048) in a replication cohort of 3308 individuals. None of these 20 suggestive sites were close to either of the associated sites observed in the current study. To reach replicable findings for CpG sites associated with MDD it is likely that larger sample sizes will be needed as has been demonstrated in genome-wide associations studies (30).

The results reported here are based on a single European population and their applicability to other countries and ancestries is unknown. GS:SFHS is a family-based sample drawn from the general population. Therefore, the more extreme phenotypes that have been analysed in the published literature would not have provided adequate power in the studied cohort. Ascertainment bias may also be present in GS:SFHS; for example, preterm birth can compromise normal neurodevelopment and increase an individual’s risk of chronic disease in later life (61), which in turn may limit participation in the study. There was also no measure of childhood abuse or neglect collected in GS:SFHS and those phenotypes may provide additional avenues for investigation using a similar methodology to that used here. Studies with larger sample sizes have reported phenotypic associations between the early life variables and mental health; however, in GS:SFHS many of these associations were not significant. This suggests that the current study was underpowered, there are differences in the variables examined, other studies were false positives or a combination of those factors. Finally, the DNA methylation data analysed was obtained from blood and the analysis of other tissue samples may reveal additional associations with the phenotypes examined.

In conclusion, there were plausible CpG sites associated with preterm birth, low birth weight and having a young parent in both MWAS 1 and MWAS 2. Further, one of the more interesting findings was the association between birth date and blood cell type composition for neutrophils and lymphocytes. It was not possible to predict either MDD or BRS from methylation profile scores calculated from early life phenotypes. Although, there was significant predictive ability of the methylation profile scores across the two sets of GS:SFHS data for birth month and having a young parent for their respective phenotypes.

## Materials and Methods

### Generation Scotland: Scottish Family Health Study (GS:SFHS)

GS:SFHS (26) is a family-based cohort study of 24 080 participants (14 154 female and 9926 male) aged between 18 and 100 (mean = 47.6 years, standard deviation [SD] = 15.4 years). Baseline data were collected between 2006 and 2011 and covered medical, behaviour and lifestyle factors with a subset recontacted (*N* = 9618) in 2015 and 2016 with additional phenotypes collected (62). Clinical information from linked electronic hospital records across the life course was also available. At the baseline appointment, a blood draw was taken from each participant, which has so far been used to obtain DNA methylation data for 9773 individuals.

### Phenotypes

Multiple phenotypes were generated from the baseline and recontact data focused on either early life environments or adult mental health. The early life environments were broadly categorized as either biological (preterm birth, low birth weight, birth date and birth month) or sociodemographic (having a lone parent, having a young parent, urbanicity and population density). The adult mental health measures were MDD and psychological resilience (measured using the BRS). The demographics of GS:SFHS are provided in Table 3.

**Table 3:** Demographic information for Generation Scotland: Scottish Family Health Study

	Whole sample			Methylation sample		
Item	*N*	Present/absent (%)	Mean, SD	*N*	Present/absent (%)	Mean, SD
Female	24 080	14 154/9926 (58.8)		9537	5638/3899 (59.1)	
Age	24 080		47.64, 15.41	9537		49.81, 13.70
Preterm birth	3134	109/3025 (3.5)		950	31/919 (3.3)	
Low birth weight	3129	103/3026 (3.3)		949	30/919 (3.2)	
Birth month	24 080	12 842/11 238 (53.3)		9516	5459/4057 (57.4)	
Birth date	22 656		0.01, 0.71	9509		0.01, 0.71
Young parent	23 546	1241/22 305 (5.3)		9398	534/8864 (5.7)	
Lone parent	22 543	1333/21 210 (5.9)		9086	580/8506 (6.4)	
Urban environment	19 428	11 739/7689 (60.4)		8017	5468/2549 (68.2)	
Population density	19 426		1949.72, 1611.32	8017		2019.44, 1489.35
Major depressive disorder	21 340	2766/18 574 (13.0)		9481	1624/7857 (17.1)	
Brief resilience scale	9354		0.00, 3.80	4839		−0.19, 3.90

*Notes:* Number of individuals (*N*) available for each item. The present *N*, absent *N* and prevalence (%) are reported for binary items with mean and SD reported for continuous items.

Age was reported in years, a preterm birth was recorded as present for those born < 37 weeks gestation length, a low birth weight was recorded as present for individuals born in the 3rd centile for birth weight adjusted for sex and gestation length, birth month compares those born between April and October inclusive (present) with those born the rest of the year (absent), birth date is a continuous measure of when an individual was born during the year with a maximum value for those born 21st June and a minimum value for those born 21st December, a young parent was present for individuals when either parent was <21 years old when the individual was born, a lone parent was recorded as present for individuals who were not living with both parents when the individual was born, an urban environment was present for individuals recorded as living in Edinburgh, Glasgow, Aberdeen or Dundee when they were born, population density was the number of individuals per square kilometre in their region when an individual was born, MDD status was based on a structured clinical interview and brief resilience scale was a continuous measure of an individual’s psychological resilience.

Preterm births were categorized as a recorded gestation of period of <37 weeks [based on World Health Organization guidance (63)] using the SMR02—Maternity Inpatient and Day Case linked electronic health records. These records were available for individuals born after 1992. Birth weight for GS:SFHS participants was also obtained from SMR02 electronic health records. The threshold for low birth weight was based on the sex and gestation length adjusted 3rd centile for birth weight reported using a Scottish sample and SMR02 records by Bonellie *et al.* (64). Preterm birth and low birth weight were assessed as binary traits following Nosarti *et al.* (9) and Colman *et al.* (65).

Seasonality of birth was assessed using two phenotypes. First, a binary phenotype for birth month was generated with those born between April and October inclusive compared with those born during the remaining months. These months were selected based on the review of birth month and depression by Schnittker (12), with increased risk of depression reported for those born April through October. Second, the birth date during the year was assessed as a continuous phenotype ($y$) and calculated as:

$$ y=-1\times \mathit{\cos}\left(2\pi \left(\frac{\mathrm{date}+10}{365}\right)\right) $$

where $y$ ranged from −1 for those born on the winter solstice (21st December) to +1 for those born on the summer solstice (21st June). $\mathrm{date}$ was the day of birth during the year for each participant (January 1st = 1, January 2nd = 2, etc.). For those born on the 29th February, $\mathrm{date}$ = 59.5.

Using the self-reported age of an individual’s parents at the time of birth, an individual was classified as having a young parent if either parent was under the age of 21. Where an individual was recorded as having a lone parent (see below), the age of the parent that the individual lived with was used.

An individual was recorded as having a lone parent if either their mother or father was recorded as living in a different country or region to the individual and the other parent at the time of birth of the individual. The regions reflect the 32 council areas of Scotland. Individuals with missing information, or where an individual was reported not to live with either parent, were excluded.

The region that an individual was living in at the time of their birth was also used to define an urban environment phenotype. Individuals living in Edinburgh, Glasgow, Aberdeen or Dundee were classified as being urban and those living in other regions as non-urban. The year of an individual’s birth and the region they were living in were also used to obtain a measure of population density based on population estimates from the National Records of Scotland (66) and was recorded as the number of individuals per square kilometre.

To determine MDD status, the initial screening questions from the Structured Clinical Interview for DSM-IV (SCID) Non-Patient Version (67) were used to identify those individuals that would subsequently complete the mood sections of the SCID. The SCID was administered by nurses trained in its application and further information on the MDD criteria used in GS:SFHS is reported in Fernandez-Pujals *et al.* (68). Participants who met the criteria for at least one MDD episode in the mood sections of the SCID were classified as cases and those who did not meet this criterion or did not report MDD symptoms in the initial screening were classified as controls.

The BRS (69) was used to determine a continuous measure of an individual’s psychological resilience and ability to ‘bounce back’ from stressful events. BRS was obtained for a subset (*N* = 9505) of the cohort during a recontact of all GS:SFHS participants in 2015 and 2016. BRS was ascertained from the response to six questions using a 5-point Likert scale with further details on the assessment provided by Navrady *et al.* (62). Individuals that responded to five or more questions were retained (*N* = 9354) with 55 missing values imputed using the missForest package (70) applying 500 trees per forest. A principal component analysis using the Psych package (71) was applied to the data to extract the first unrotated principal component which was then scaled to create a continuous measure of BRS.

### Regression of adult mental health on early life environments

To determine whether there was an association between early life stressors and the measures of adult mental health a generalized linear mixed model was used from the lme4qtl package (72). MDD and BRS were assessed separately as the dependent variable for an association with each early life factor in turn as the independent variable. The total genetic value derived from pedigree data [with the variance/covariance structure defined by a kinship matrix using the kinship2 package (73)] was fitted as a random effect, jointly with sex fitted as a fixed effect. Binary traits were fitted as factors and continuous traits were centred and scaled to have a mean of 0 and a SD of 1. A binomial regression with a logit function was used to assess associations with MDD. For a significant association between a mental health trait and an early life environment a Bonferroni correction was applied to the *P*-values within each mental health trait, *P* < 6.25 × 10^−3^ (α = 0.05/8).

### Methylation data

The Infinium MethylationEPIC BeadChip (Illumina Inc., USA) was used to profile DNA methylation data at 853 307 CpG sites generated in two sets of individuals. There were 5190 individuals in Set 1 and 4583 unrelated individuals in Set 2, with no related individuals between the sets. Quality control was then applied to both Sets and full details are provided by Barbu *et al.* (60) and McCartney *et al.* (74). In summary, individuals were excluded if they were outliers based on multidimensional scaling, the predicted sex from the methylation data mismatched the recorded sex, or where ≥1% of CpG sites had a detection *P*-value > 0.05. CpG sites were excluded where (i) the beadcount was <3 in >5% of individuals or (ii) where sites in which ≥0.5% of individuals had a detection *P*-value > 0.05. The sites identified as non-specific binding and/or polymorphisms at the target site by McCartney *et al.* (75) were removed. The log2 ratio of the intensities of methylated probe versus unmethylated probe data was used to generate methylation *M*-values separately in each Set (76). In total there were 713 522 CpG sites remaining with 5087 individuals in Set 1 and 4450 individuals in Set 2.

After combining the Sets (9537 individuals), correction was applied for:

(1) Technical variation, where *M*-values were included as dependent variables in a mixed linear model adjusting for the plate used to profile the DNA methylation data and the date of the individual’s blood draw as random effects, jointly with plate position, Set, clinic, year of appointment, day of week of appointment and the first 10 principal components (from the EPIC array control sites) as fixed effects; and
(2) Biological variation by fitting variables of (1) as dependent variables in a second mixed linear model adjusting for genetic and common family shared environmental contributions [classed as G: common genetic; K: kinship; F: nuclear family; C: couple and S: sibling, see Xia *et al.* (77) and Zeng *et al.* (78) for further information] as random effects, jointly with sex, age and estimated blood cell type composition [CD8T, CD4T, NK, Bcell, Mono, Gran; obtained using the Houseman algorithm (79) within the Meffil package (80)] as fixed effects.

### MWAS of early life environments and adult mental health

Two methods (MWAS 1 and MWAS 2) were used to conduct the MWAS generating two sets of summary statistics for each phenotype. MWAS 1 was conducted using the eBayes function within the limma package (81), which applies a linear mixed model to the data. The M-values, corrected for technical and biological variation, were the dependent variable with each phenotype included separately as the independent variable. Set, smoking status (ever or never), smoking pack years, and the first 20 principal components created from the M-values using the FactoMineR package (82), were fitted as fixed effects.

MWAS 2 was conducted using the software tool OmicS-data-based Complex trait Analysis (OSCA) (83). First, to account for methylome-wide correlational structure, this tool uses a linear regression analysis to identify groups of lead CpG sites based on the association test statistics. Each phenotype in turn was the dependent variable with the corrected *M*-values as the independent variable adjusted for the same covariates used in MWAS 1 (Set, smoking status, smoking pack years, and the first 20 principal components). Second, each group of lead sites were fitted as random effects in a Multi-component MLM-based association excluding the target (MOMENT) analysis to assess the effect of each target probe in turn, with the respective phenotype as the dependent variable and the M-values as the independent variable. MOMENT also fits predicted blood cell type composition [basophil, eosinophil, lymphocyte, monocyte and neutrophil; based on haematological analysis of the LBC (84)] as a fixed effect.

A methylome-wide significance threshold was determined using a Bonferroni correction based on the number of CpG sites analysed for each analysis: *P* < 7.01 × 10^−8^ (α = 0.05/713 522). To visualise the MWAS output, Miami plots were created using ggplot2 (85) with the *P*-values for MWAS 1 on the log_10_ scale and for MWAS 2 on the -log_10_ scale. QQ-plots were created using Haplin (86) with the shaded error representing the 95% confidence interval. Genomic inflation ($\lambda$) was calculated for each output as the median of the observed chi-squared distribution of *P*-values divided by the median of the expected chi-squared distribution.

### Gene and gene-set analysis

The significant CpG sites (*P* < 7.01 × 10^−8^) for each phenotype and each MWAS were annotated to genes based on location. For MWAS 1, the missMethyl package (87) was used to annotate genes according to location. For MWAS 2, the annotations reported by the OSCA package were used. The gometh function within missMethyl was used to analyse the data for enrichment of Gene Ontology and KEGG gene-sets. The overlap of the annotated genes and enriched gene-sets between the early life environment and adult mental health phenotypes were examined.

### Mediation analysis

During the revision of the manuscript a reviewer suggested including a mediation analysis of two CpG sites associated with birthweight and located close to *GRM8*: cg21803443 identified in the present study and cg15908975 identified by Küpers *et al.* (29). *GRM8* has previously been associated with depression (30). A mediation analysis (88) fitting birth weight as the independent variable, MDD as the dependent variable, and either cg21803443 or cg15908975 as the mediator variable was conducted.

### Methylation profile scores

MWAS 2 was used to calculate summary statistics for each early life environment phenotype using the individuals in Set 1 (*N* = 5190). Multiple *P*-value thresholds (< 10^−7^, < 10^−6^, < 10^−5^, < 10^−4^, < 10^−3^, < 10^−2^ and < 10^−1^) were used to identify those sites for inclusion. For each individual in Set 2, an aggregated methylation profile score was calculated by multiplying the CpG effect sizes from Set 1 by their respective *M*-values in Set 2. There were no related individuals between Set 1 and Set 2. The ability of these profile scores to predict the same phenotype, MDD or BRS, was assessed after adjusting for sex, age, and the first 20 principal components (derived from single nucleotide polymorphism data) as fixed effects. For prediction of binary phenotypes, a coefficient of determination was calculated using Nagelkerke’s *R*^2^ (89) using a population prevalence of that equal to the sample. For quantitative phenotypes, a simple linear regression was used with the adjusted *R*^2^ reported. A Bonferroni correction was used to identify significant prediction based on the number of early life phenotypes examined in each case: *P* < 6.25 × 10^−3^ (α = 0.05/8).

During the revision of the manuscript a reviewer suggested examining whether methylation profile scores calculated from continuous measures of preterm birth or birth weight predicted MDD or BRS. The most significant CpG site from each of the 2375 differentially methylated region identified for gestational age from Merid *et al.* (31) and the 914 CpG sites prioritized (*P* < 1.06 × 10^−7^ and *I*^2^ ≤ 50%) by Küpers *et al.* (29) for birth weight were used to calculate the respective profile score for prediction of MDD or BRS in Set 2. The same fixed effects and method for calculating *R*^2^ as described in the previous paragraph were used.

## Supplementary Material

Supplementary_Figures_ddab274

SupplementaryTable1_ddab274

SupplementaryTable2_ddab274

SupplementaryTable3_ddab274

SupplementaryTable4_ddab274

Supplementary_Information_ddab274

## Data Availability

According to Wellcome Trust’s Policy on data, software and materials management and sharing, all data supporting this study will be openly available at https://doi.org/10.7488/ds/3126.
