# Supplementary material for: Methylome-wide association study of early life stressors and adult mental health
Source: Hum Mol Genet. 2021 Sep 15;31(4):651–64. doi: 10.1093/hmg/ddab274 (PMC8863421; doi:10.1093/hmg/ddab274)
Supplement: Supplementary_Figures_ddab274 [file supplementary_figures_ddab274.docx]

Methylome-wide association study of early life stressors and adult mental health reveals a relationship between birth date and cell type composition in blood

David M. Howard et al.

Supplementary Figures


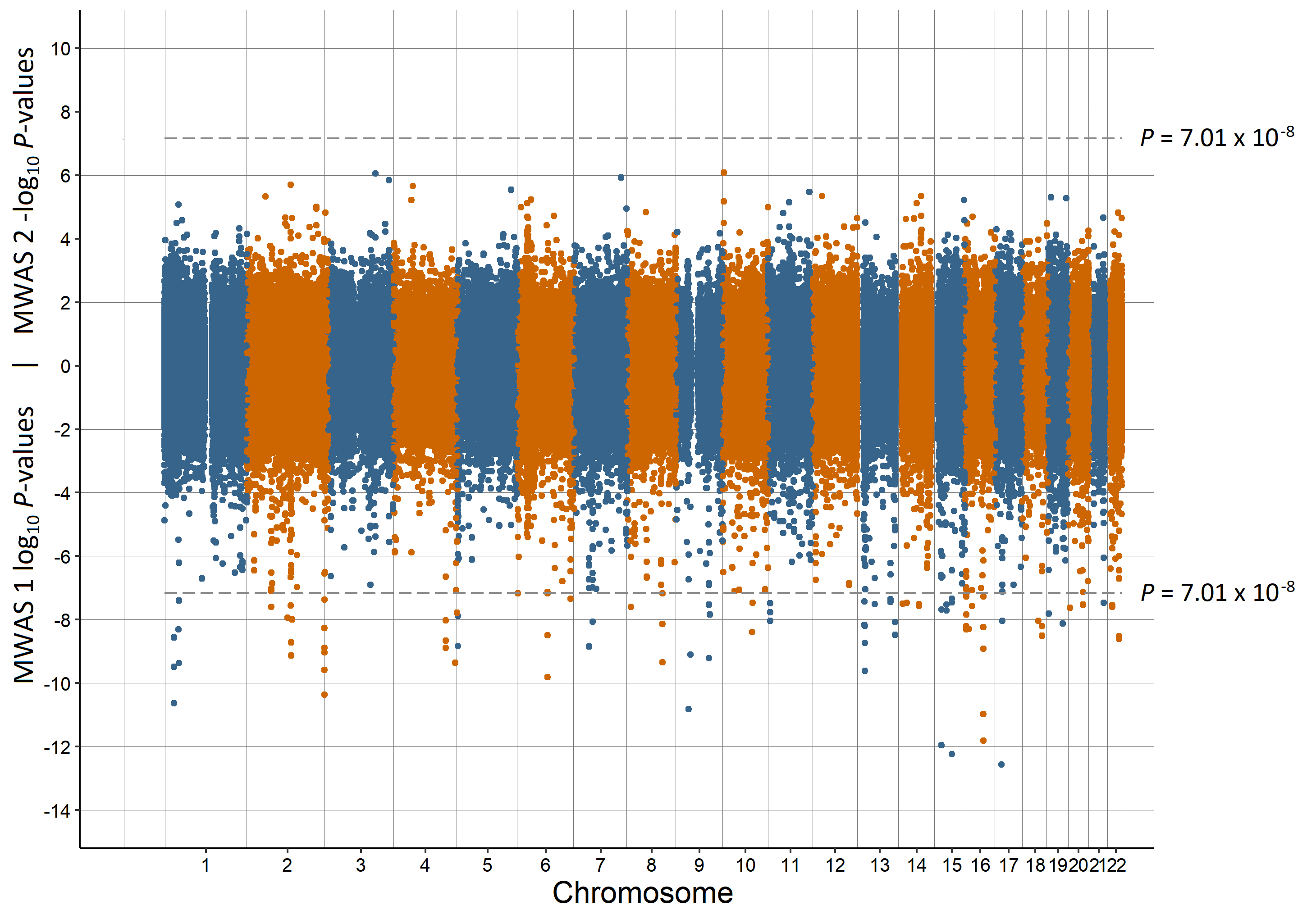


Supplementary Figure 1. Miami plot of the observed *P*-values of each CpG site for an association with birth month

Log_10_ *P*-values are shown for MWAS 1 and -log_10_ *P*-values are shown for MWAS 2. The dotted lines indicate methylome-wide significance (*P* = 7.01 × 10^-8^)


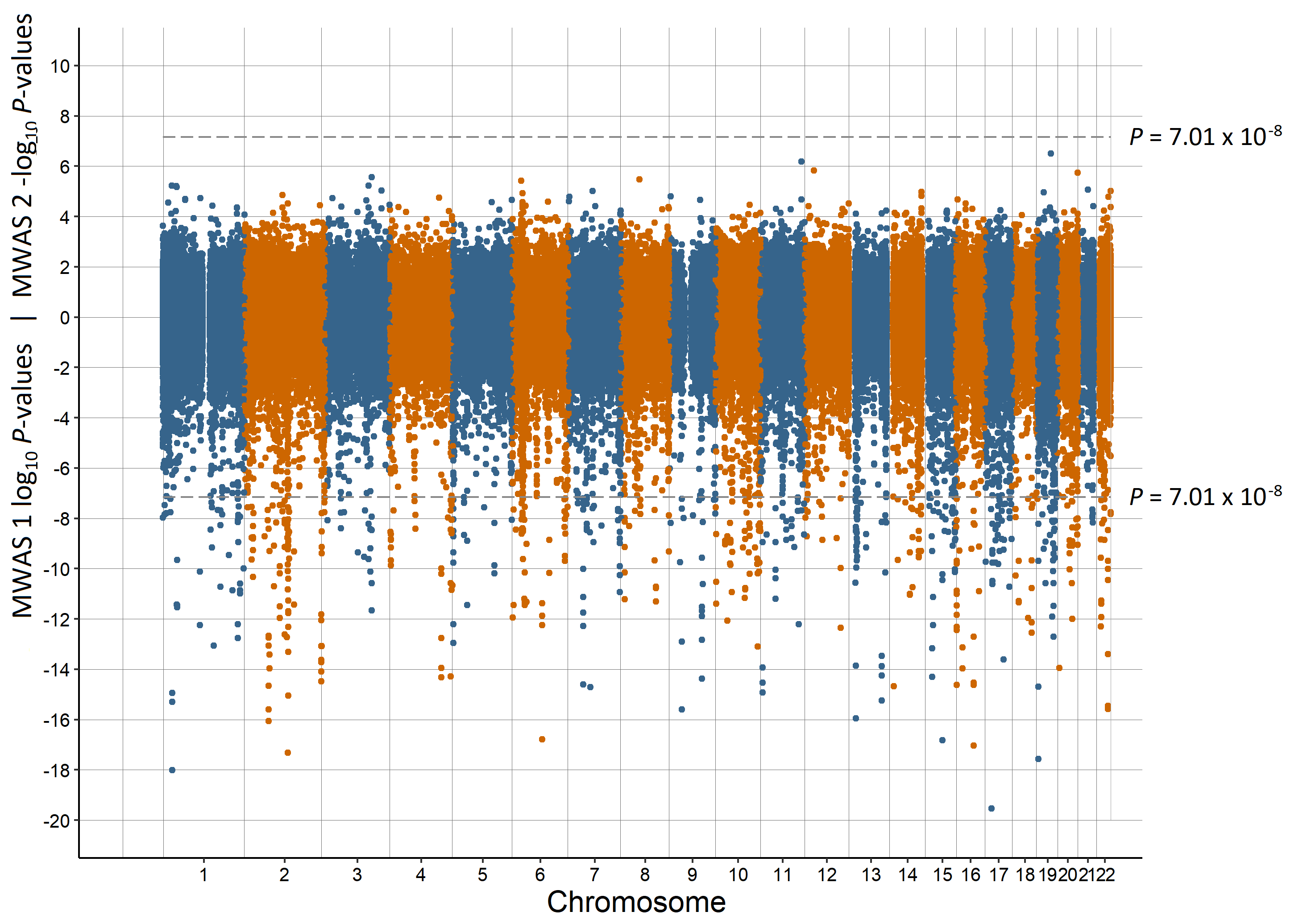


Supplementary Figure 2. Miami plot of the observed *P*-values of each CpG site for an association with birth date

Log_10_ *P*-values are shown for MWAS 1 and -log_10_ *P*-values are shown for MWAS 2. The dotted lines indicate methylome-wide significance (*P* = 7.01 × 10^-8^)


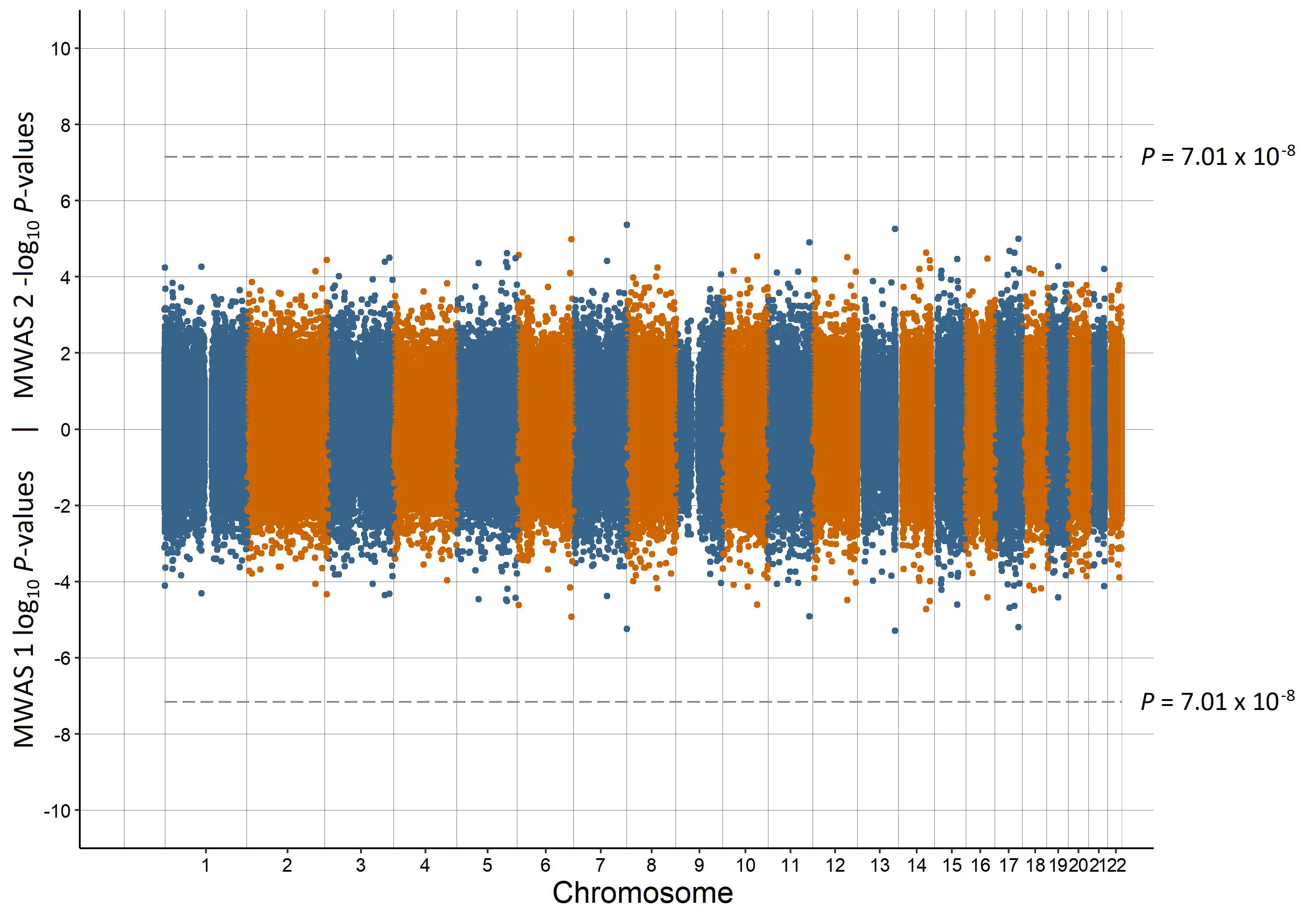


Supplementary Figure 3. Miami plot of the observed *P*-values of each CpG site for an association with having a lone parent

Log_10_ *P*-values are shown for MWAS 1 and -log_10_ *P*-values are shown for MWAS 2. The dotted lines indicate methylome-wide significance (*P* = 7.01 × 10^-8^)


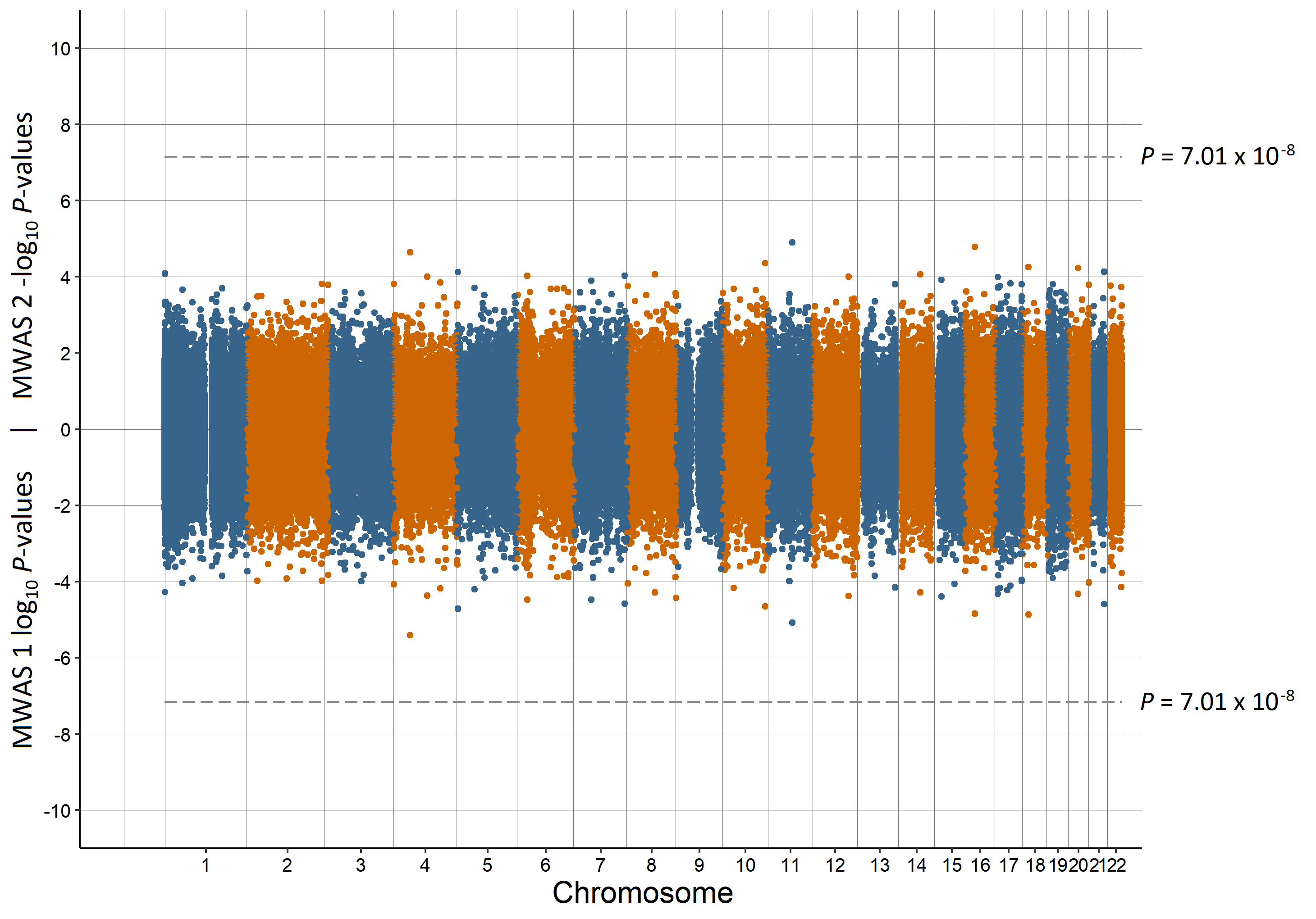


Supplementary Figure 4. Miami plot of the observed *P*-values of each CpG site for an association with urbanicity

Log_10_ *P*-values are shown for MWAS 1 and -log_10_ *P*-values are shown for MWAS 2. The dotted lines indicate methylome-wide significance (*P* = 7.01 × 10^-8^)


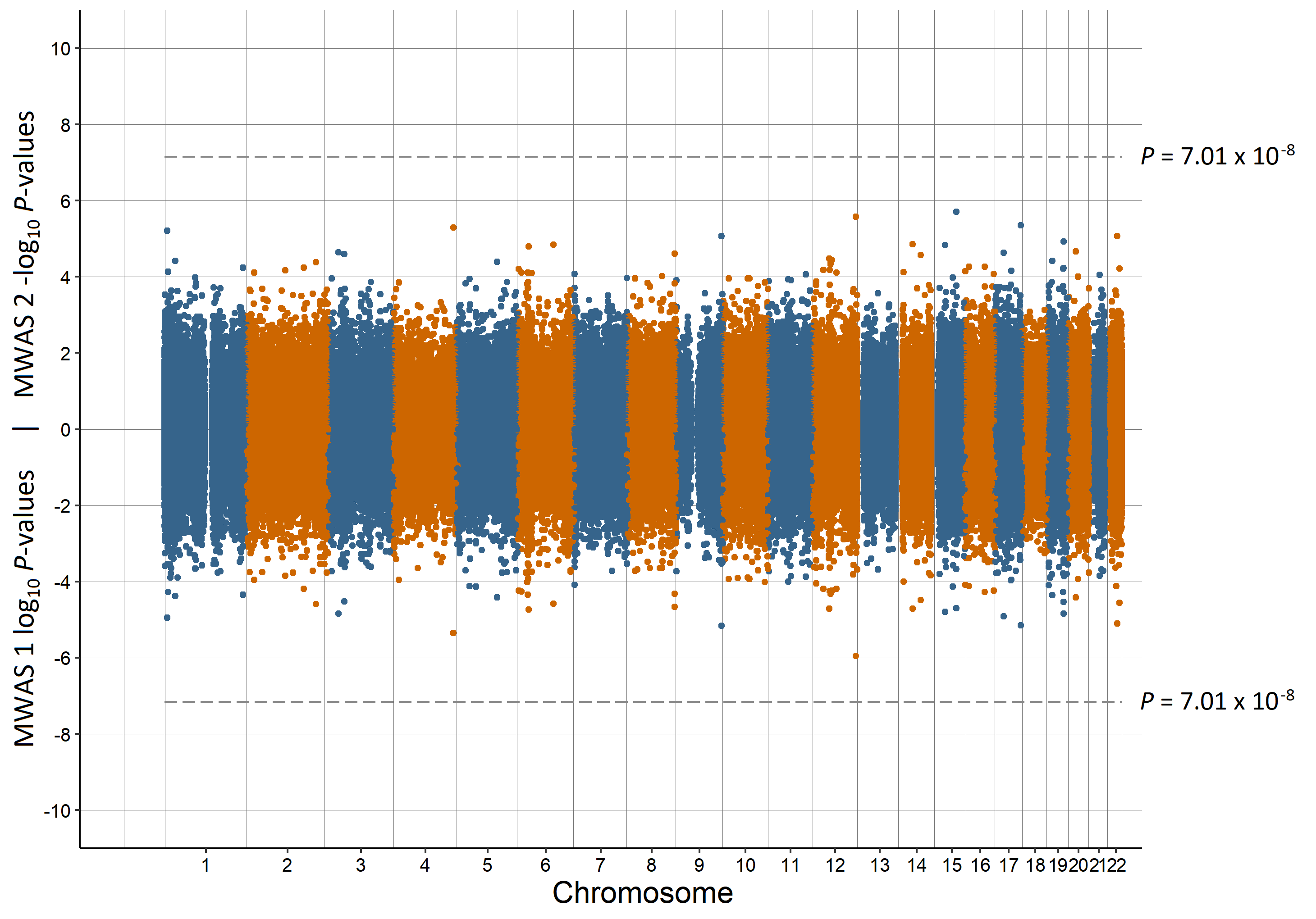


Supplementary Figure 5. Miami plot of the observed *P*-values of each CpG site for an association with brief resilience scale

Log_10_ *P*-values are shown for MWAS 1 and -log_10_ *P*-values are shown for MWAS 2. The dotted lines indicate methylome-wide significance (*P* = 7.01 × 10^-8^)


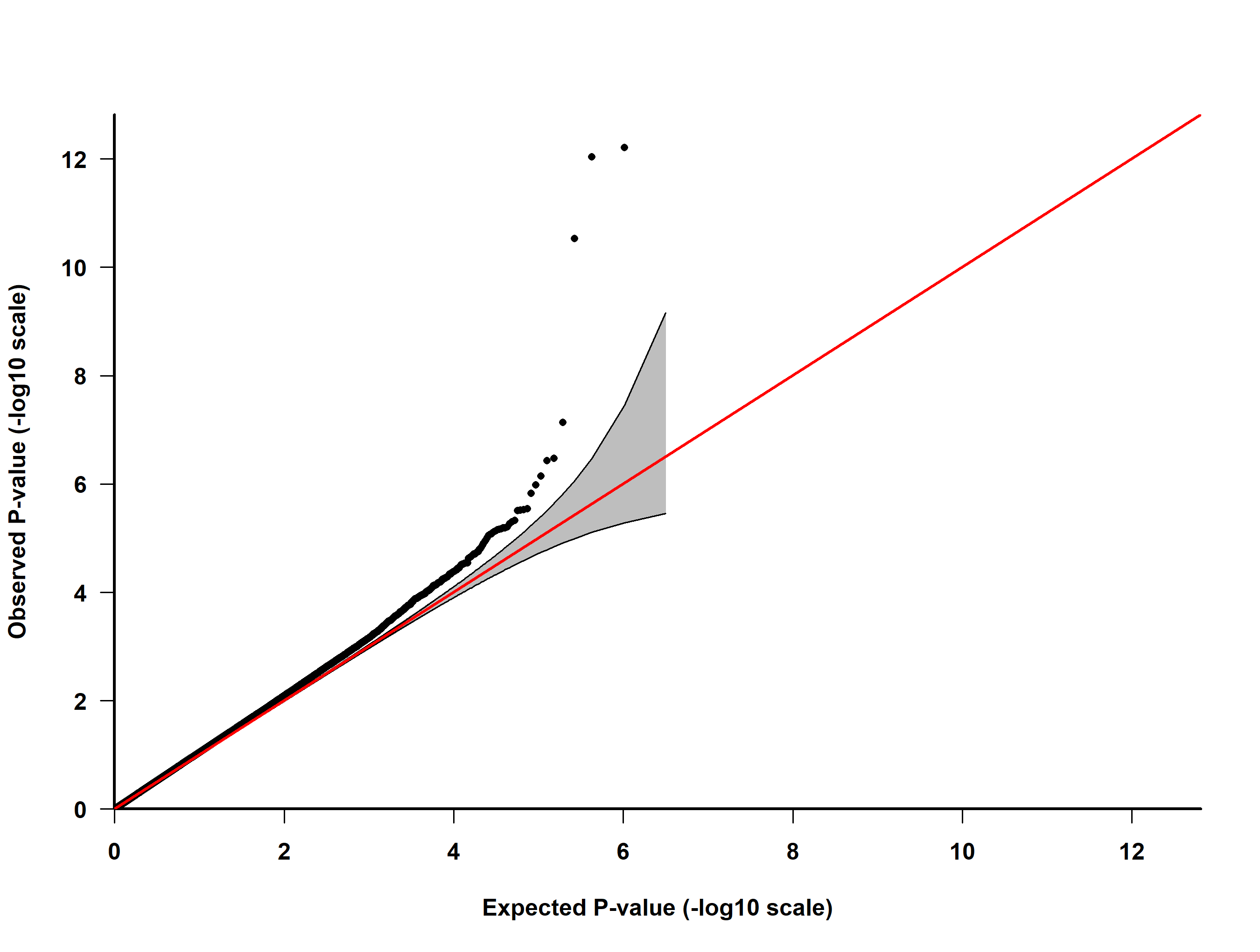

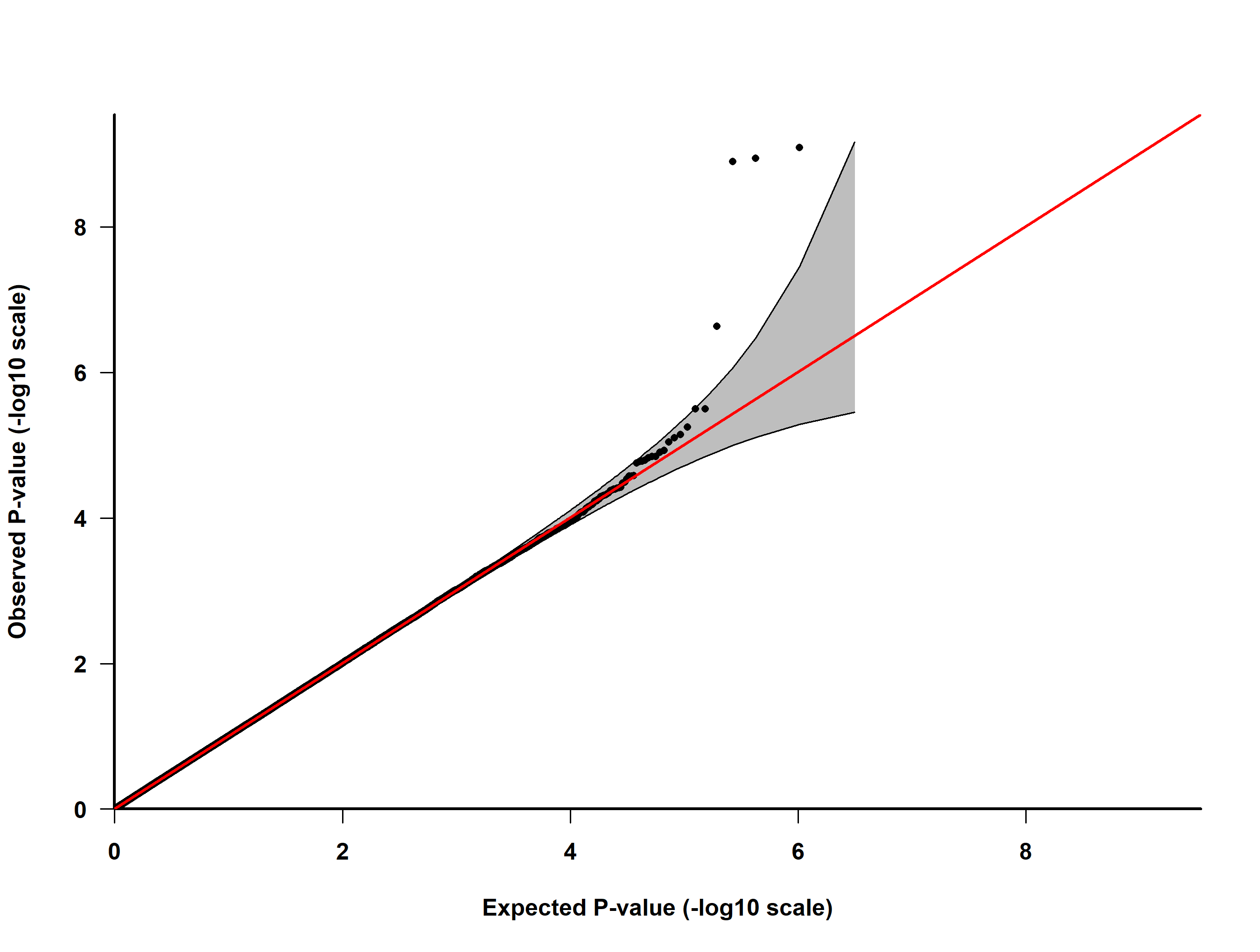


B

A

Supplementary Figure 6. QQ-plot of observed and expected *P*-values for preterm birth in MWAS 1 (A) and MWAS 2 (B)

The straight line is where the observed *P*-values match those expected and the shaded area is the 95% confidence interval. Genomic inflation: MWAS 1 = 1.070, MWAS 2 = 1.018


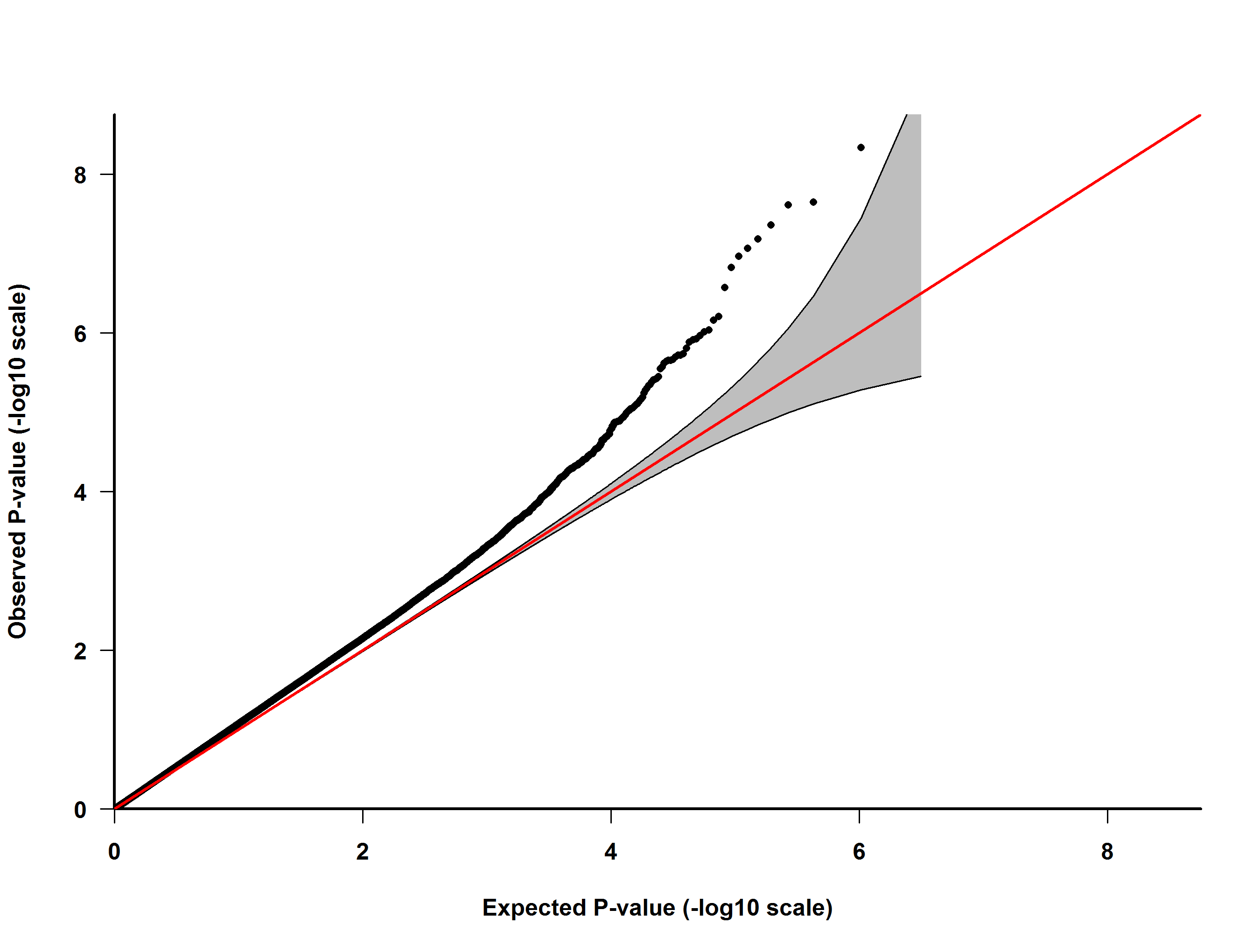

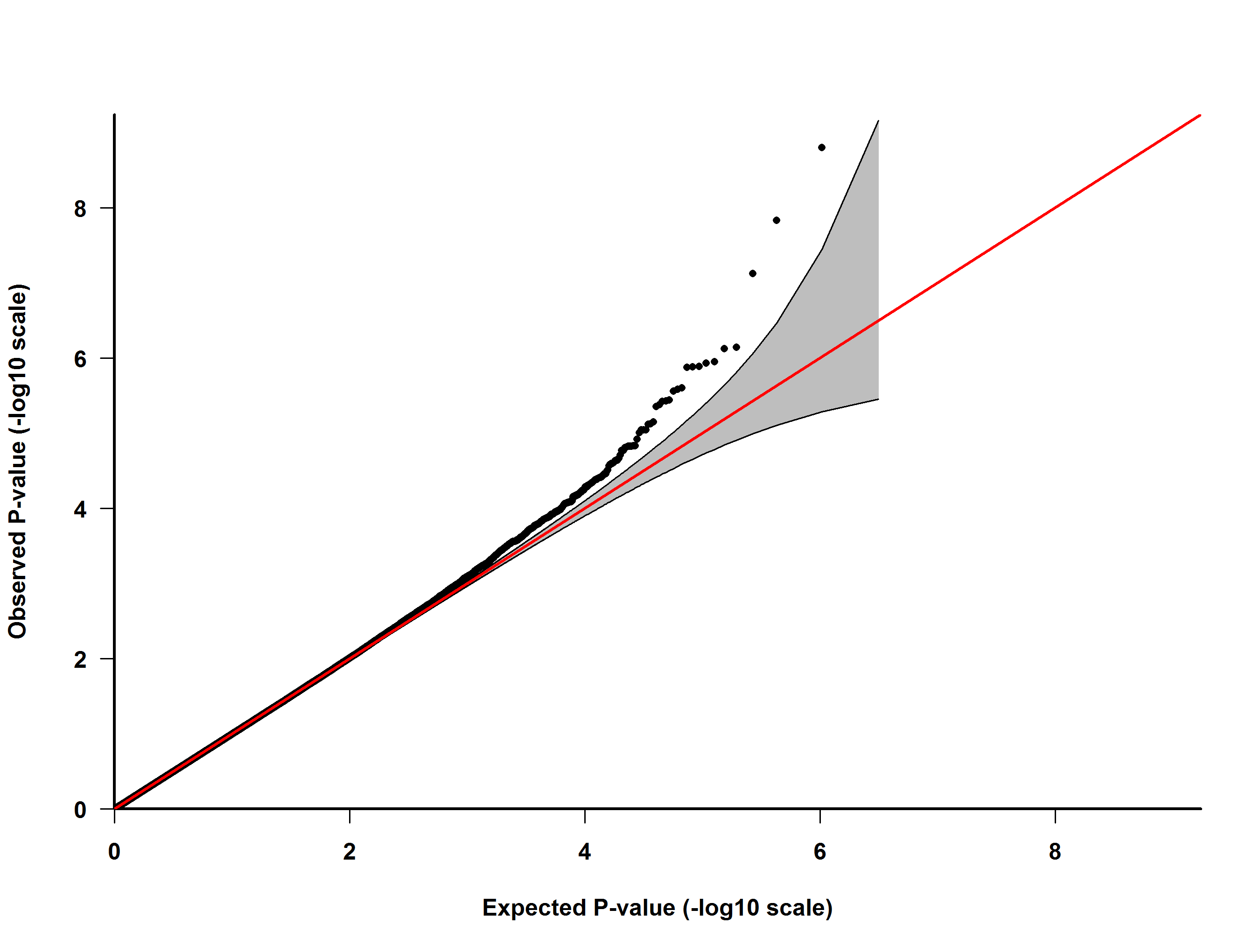


B

A

Supplementary Figure 7. QQ-plot of observed and expected *P*-values for low birth weight in MWAS 1 (A) and MWAS 2 (B)

The straight line is where the observed *P*-values match those expected and the shaded area is the 95% confidence interval. Genomic inflation: MWAS 1 = 1.117, MWAS 2 = 0.999


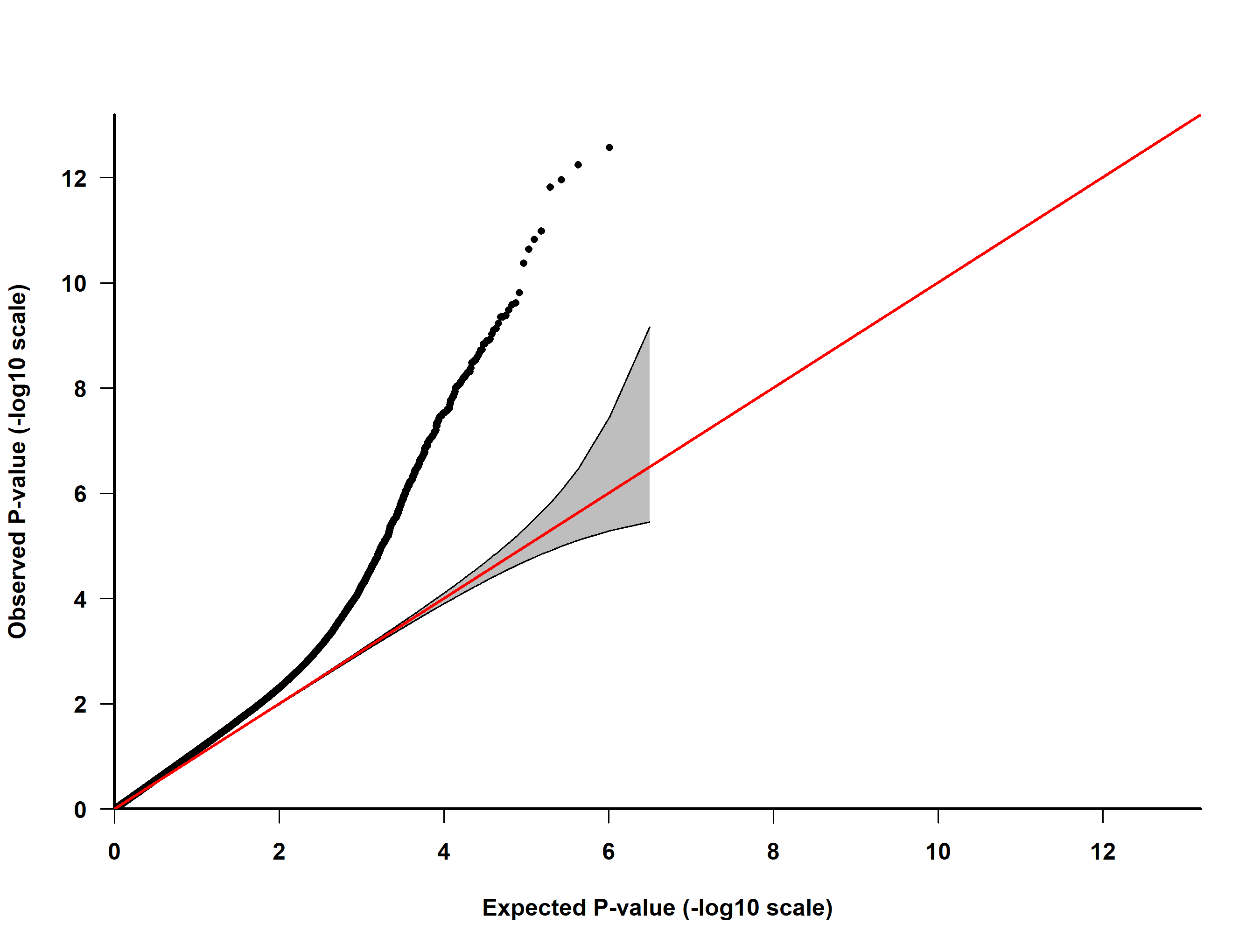

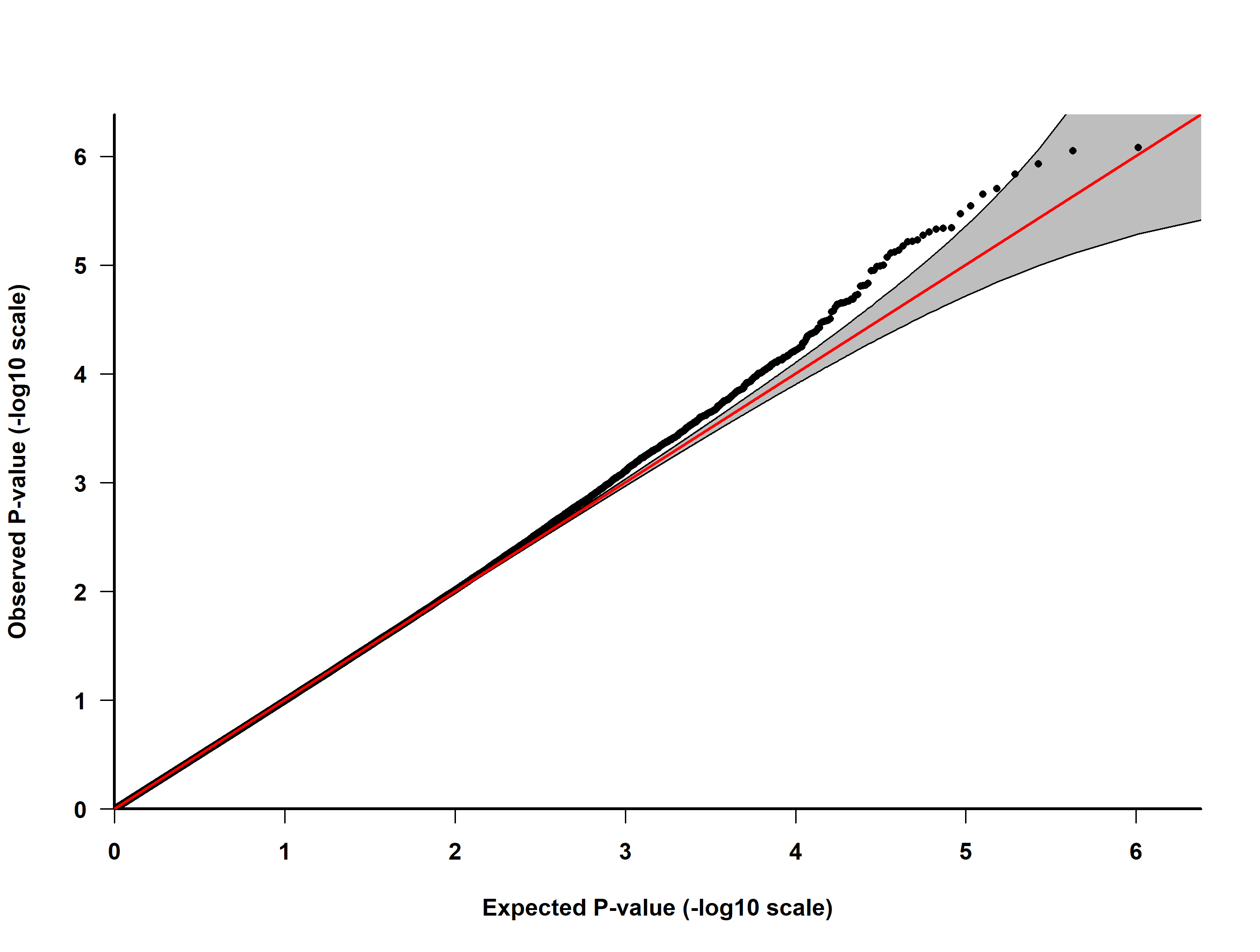


B

A

Supplementary Figure 8. QQ-plot of observed and expected *P*-values for birth month in MWAS 1 (A) and MWAS 2 (B)

The straight line is where the observed *P*-values match those expected and the shaded area is the 95% confidence interval. Genomic inflation: MWAS 1 = 1.166, MWAS 2 = 0.989


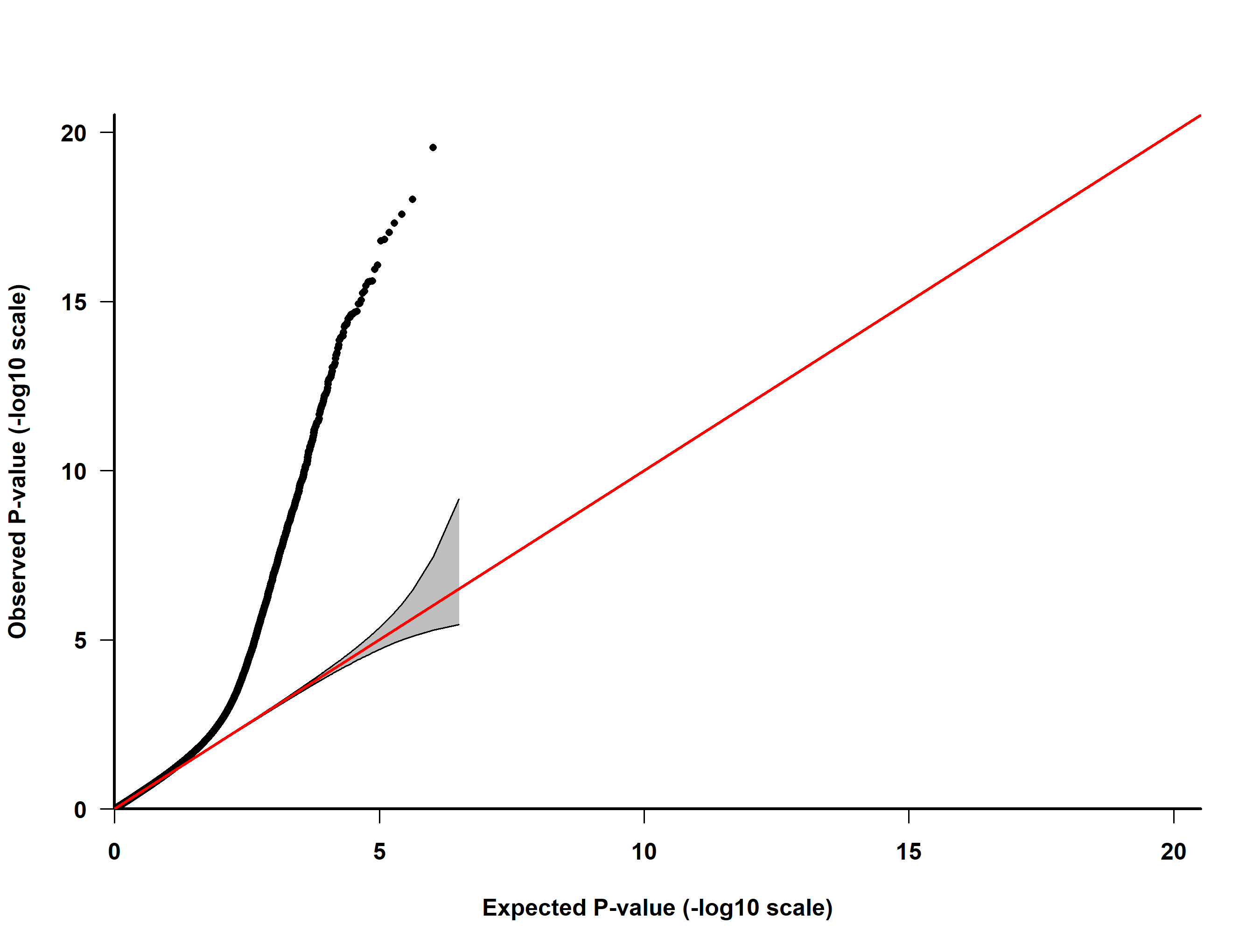

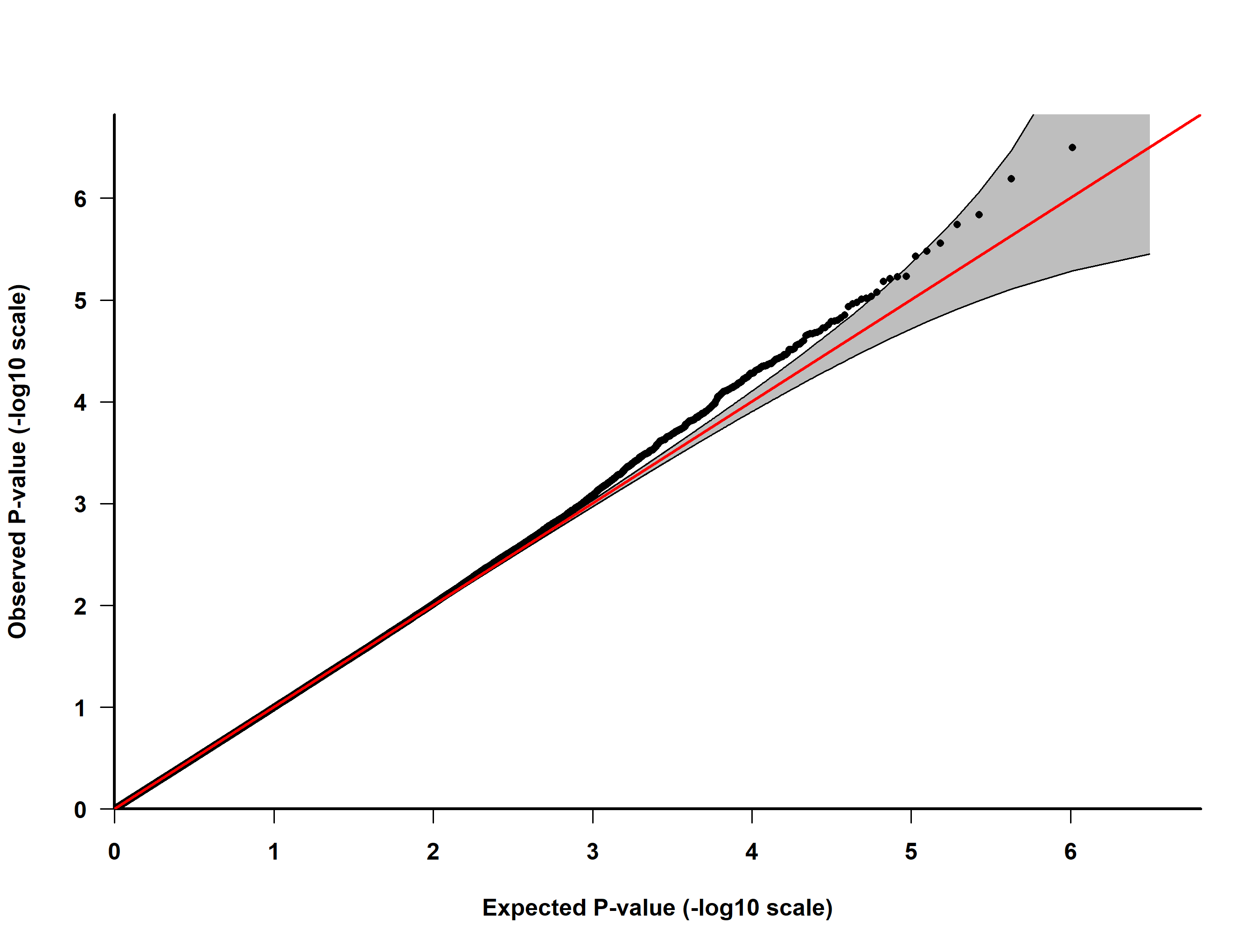


B

A

Supplementary Figure 9. QQ-plot of observed and expected *P*-values for birth date in MWAS 1 (A) and MWAS 2 (B)

The straight line is where the observed *P*-values match those expected and the shaded area is the 95% confidence interval. Genomic inflation: MWAS 1 = 1.010, MWAS 2 = 0.989


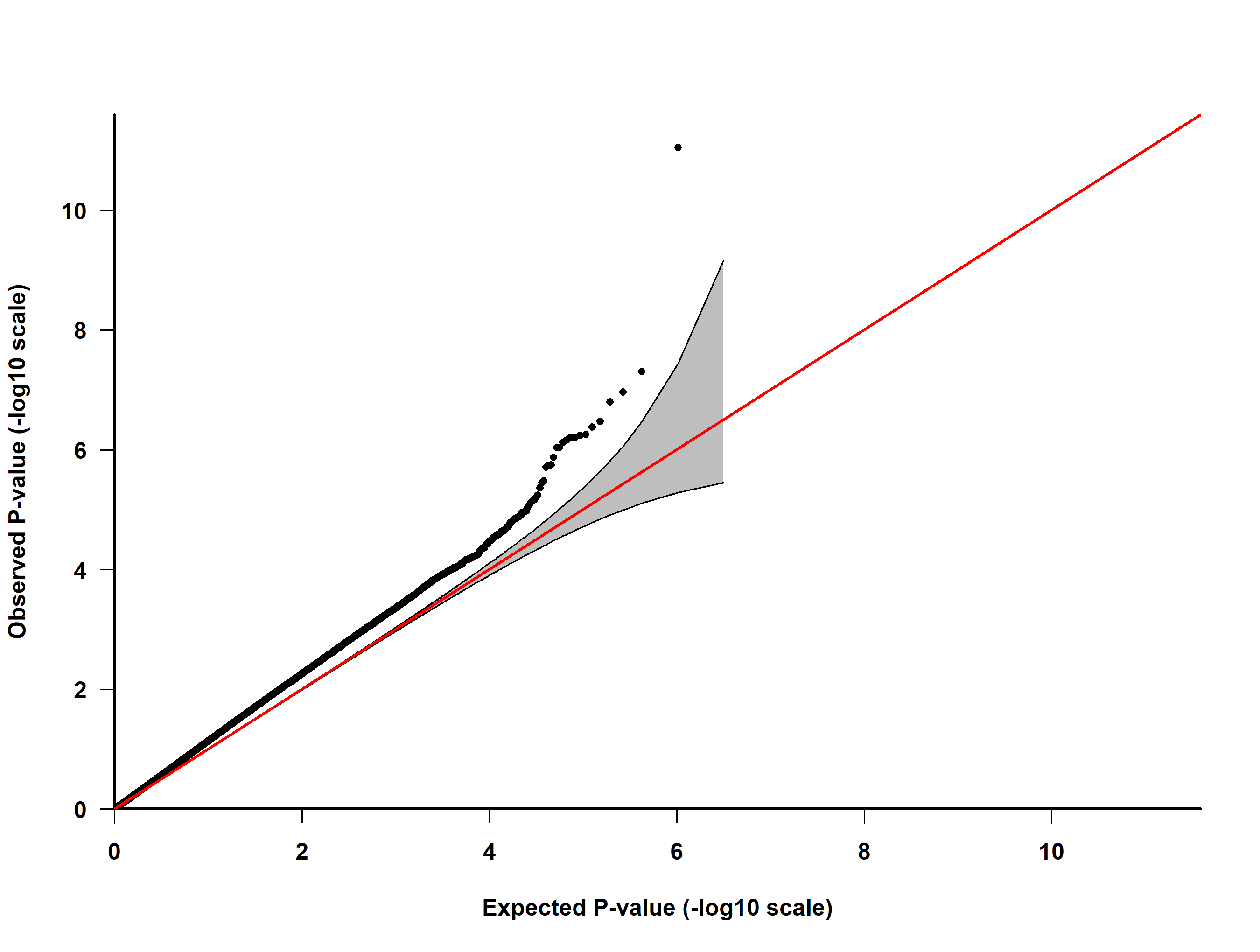

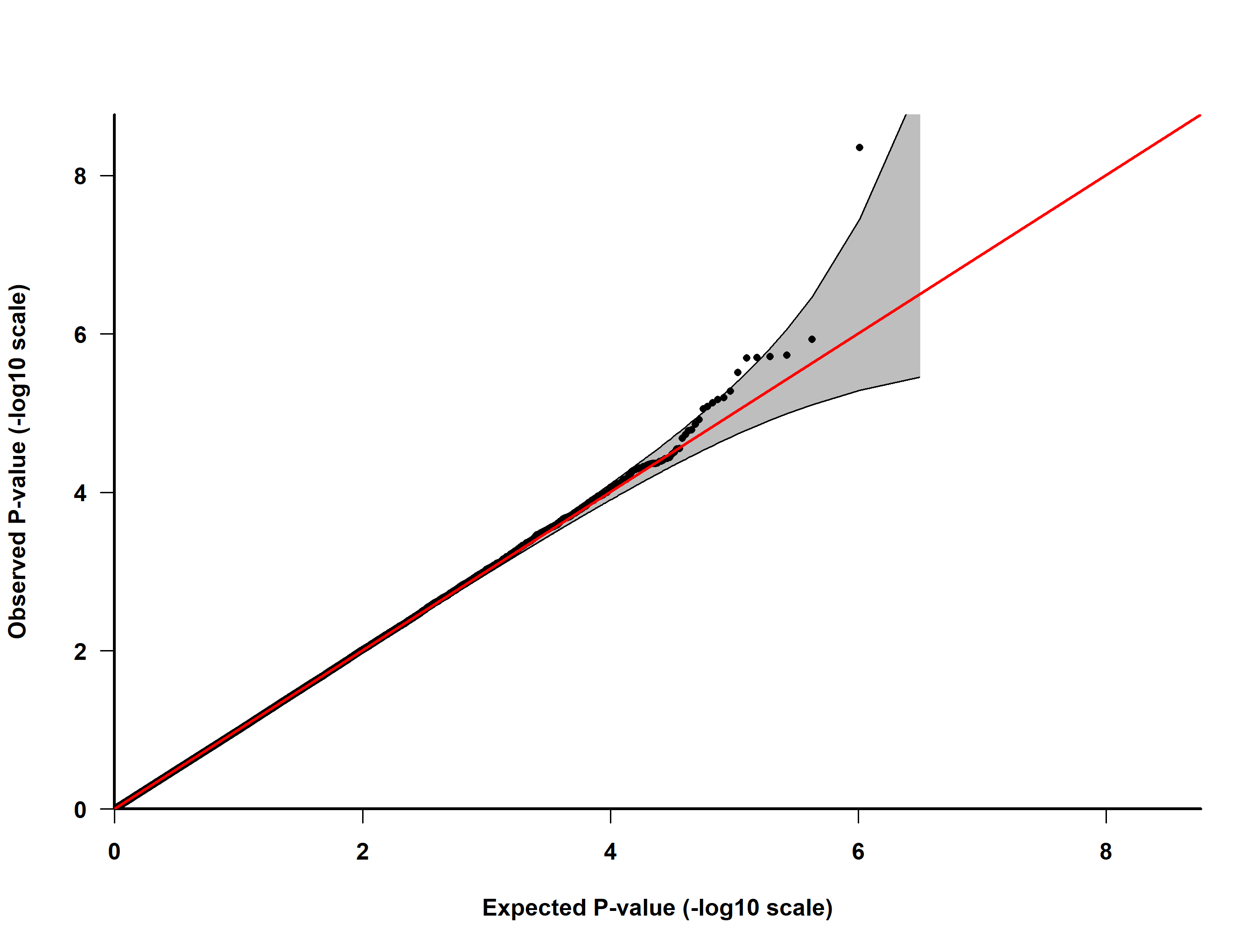


B

A

Supplementary Figure 10. QQ-plot of observed and expected *P*-values for having a young parent in MWAS 1 (A) and MWAS 2 (B)

The straight line is where the observed *P*-values match those expected and the shaded area is the 95% confidence interval. Genomic inflation: MWAS 1 = 1.207, MWAS 2 = 0.997


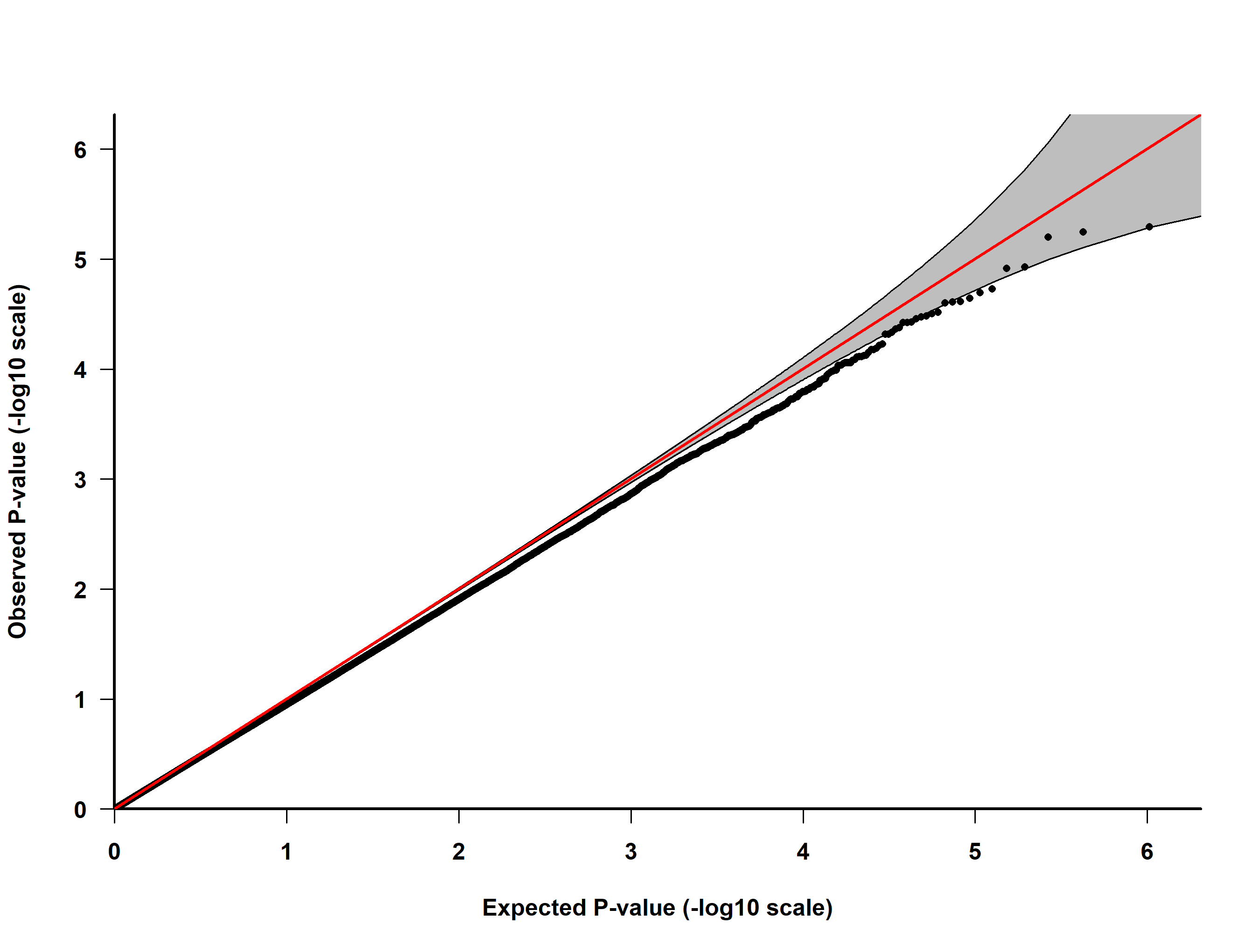

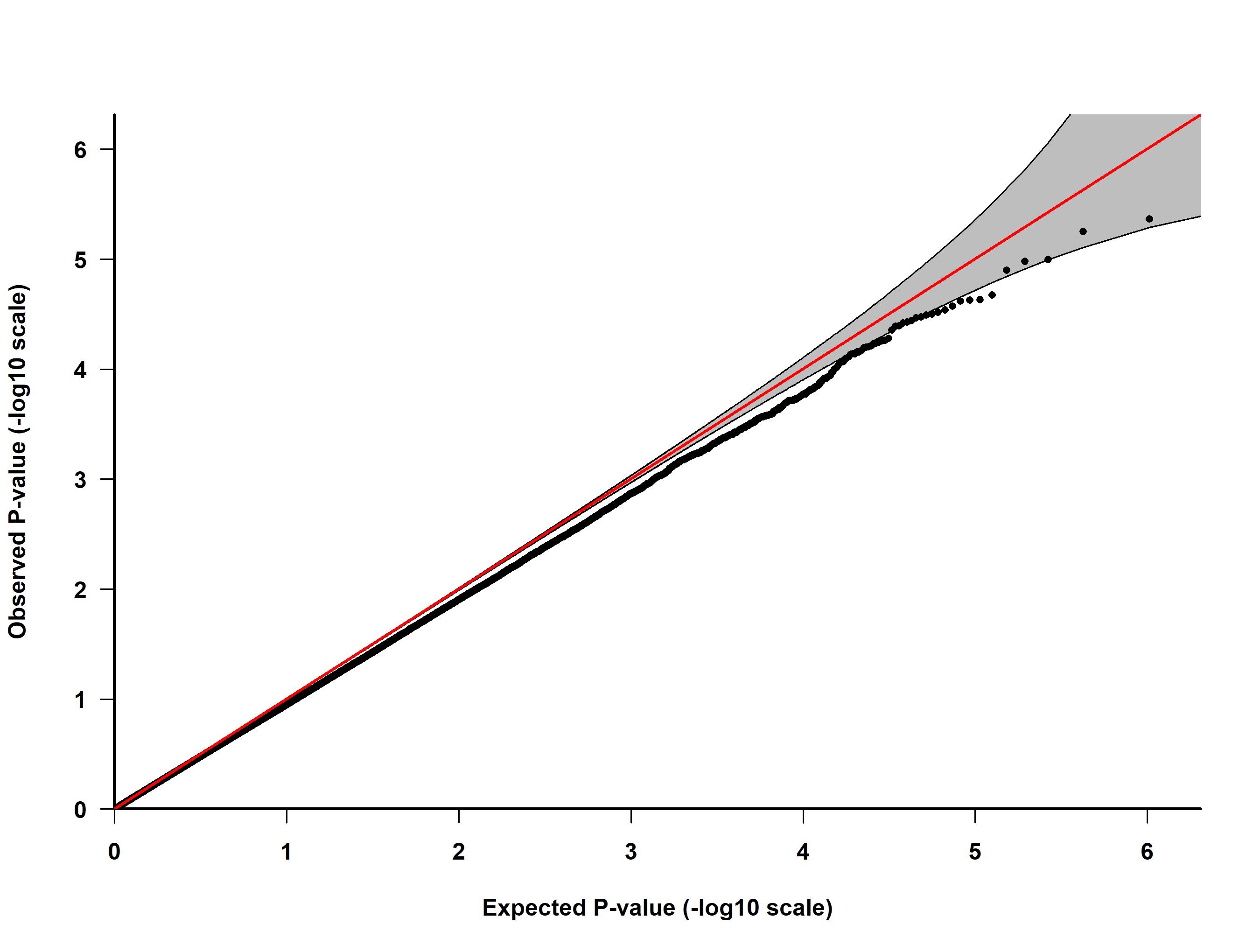


B

A

Supplementary Figure 11. QQ-plot of observed and expected *P*-values for having a lone parent in MWAS 1 (A) and MWAS 2 (B)

The straight line is where the observed *P*-values match those expected and the shaded area is the 95% confidence interval. Genomic inflation: MWAS 1 = 0.932, MWAS 2 = 0.932


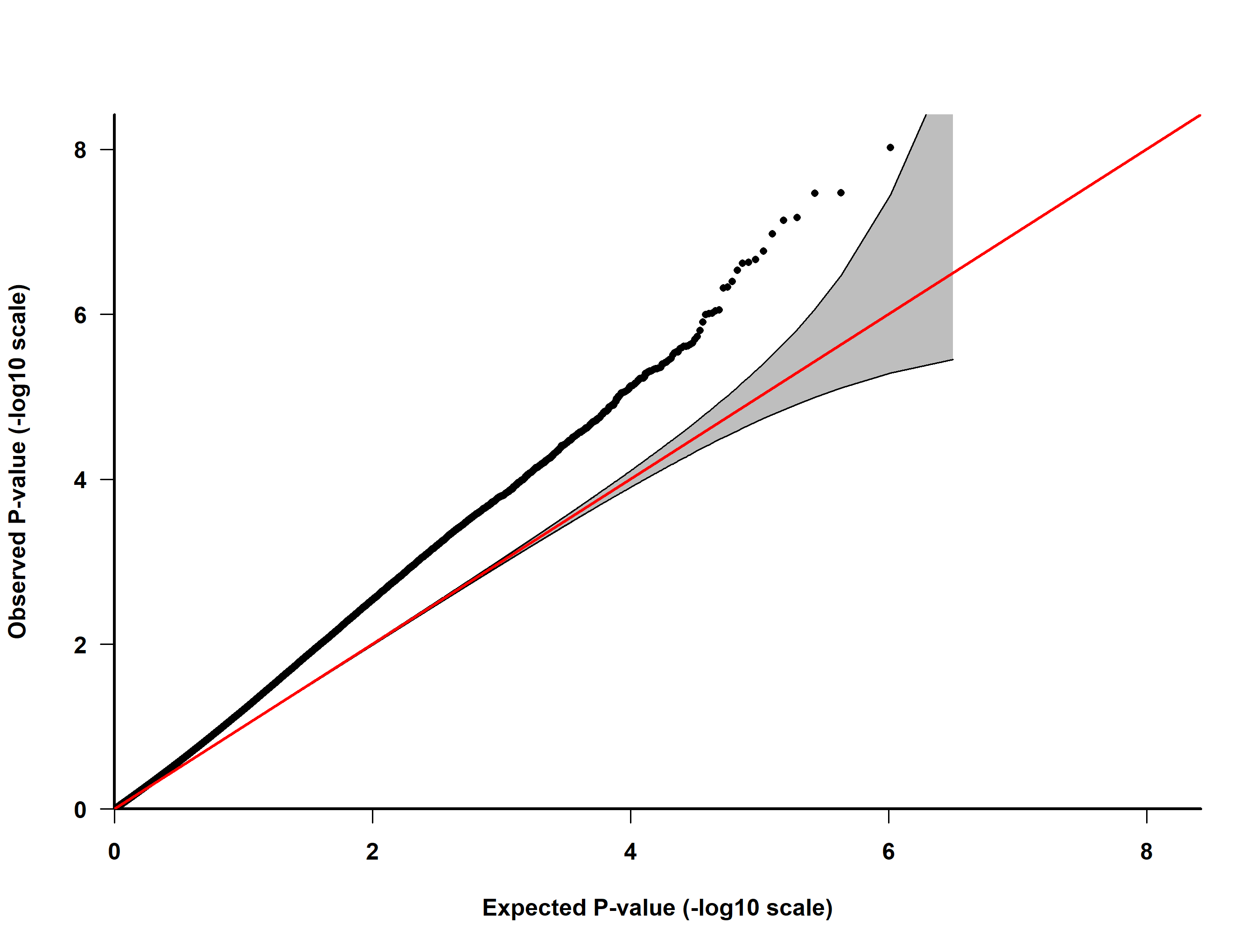

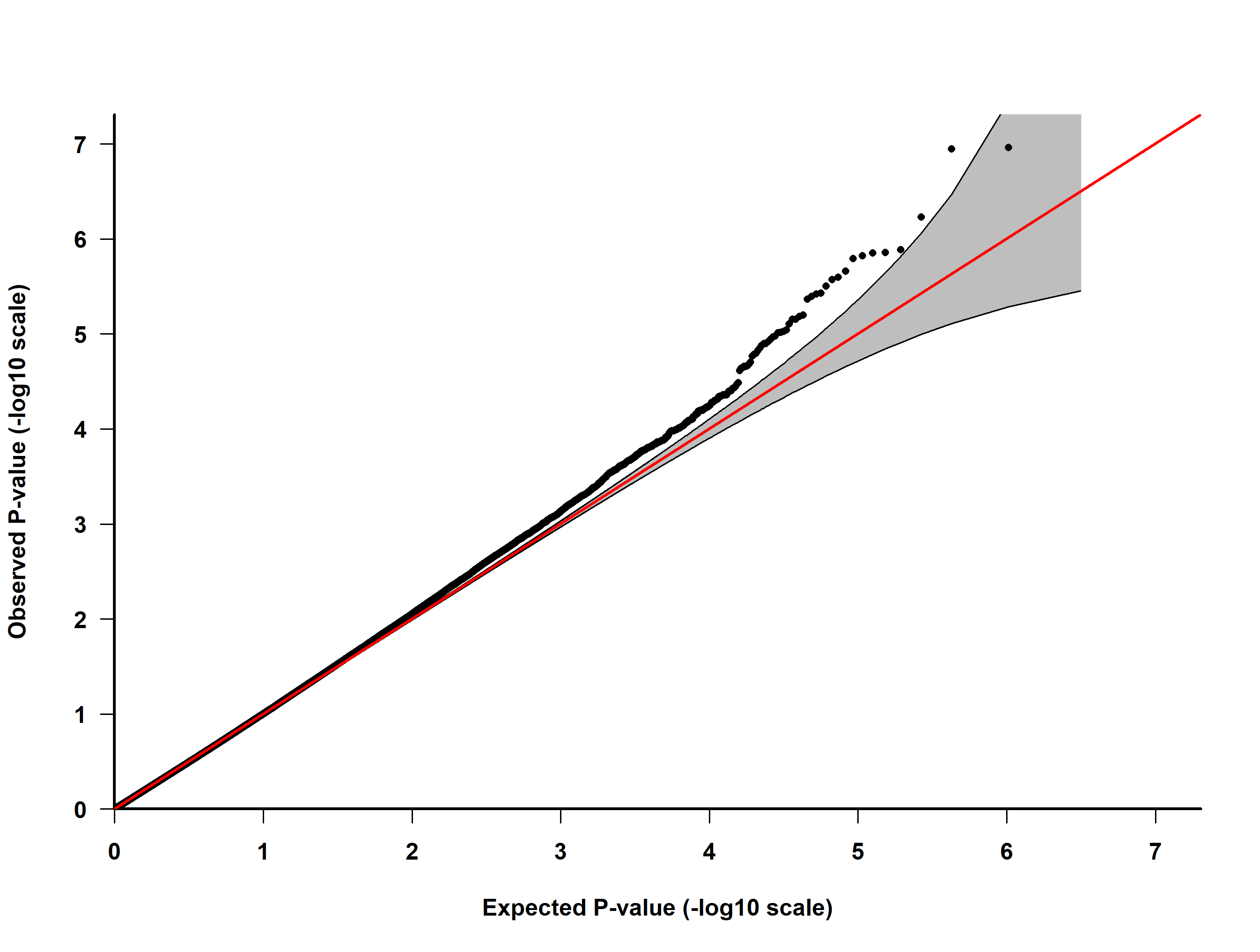


B

A

Supplementary Figure 12. QQ-plot of observed and expected *P*-values for population density in MWAS 1 (A) and MWAS 2 (B)

The straight line is where the observed *P*-values match those expected and the shaded area is the 95% confidence interval. Genomic inflation: MWAS 1 = 1.215, MWAS 2 = 0.978


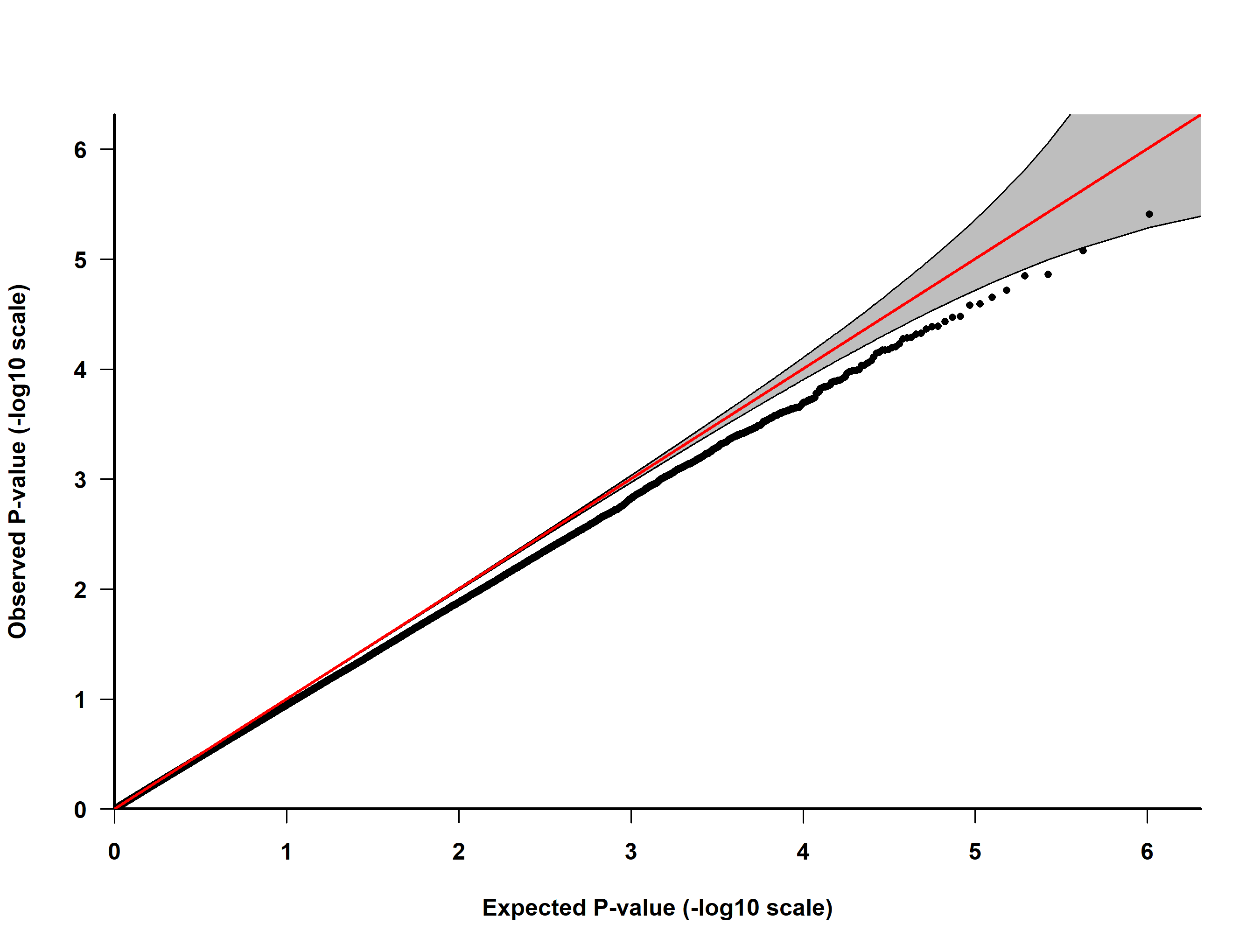

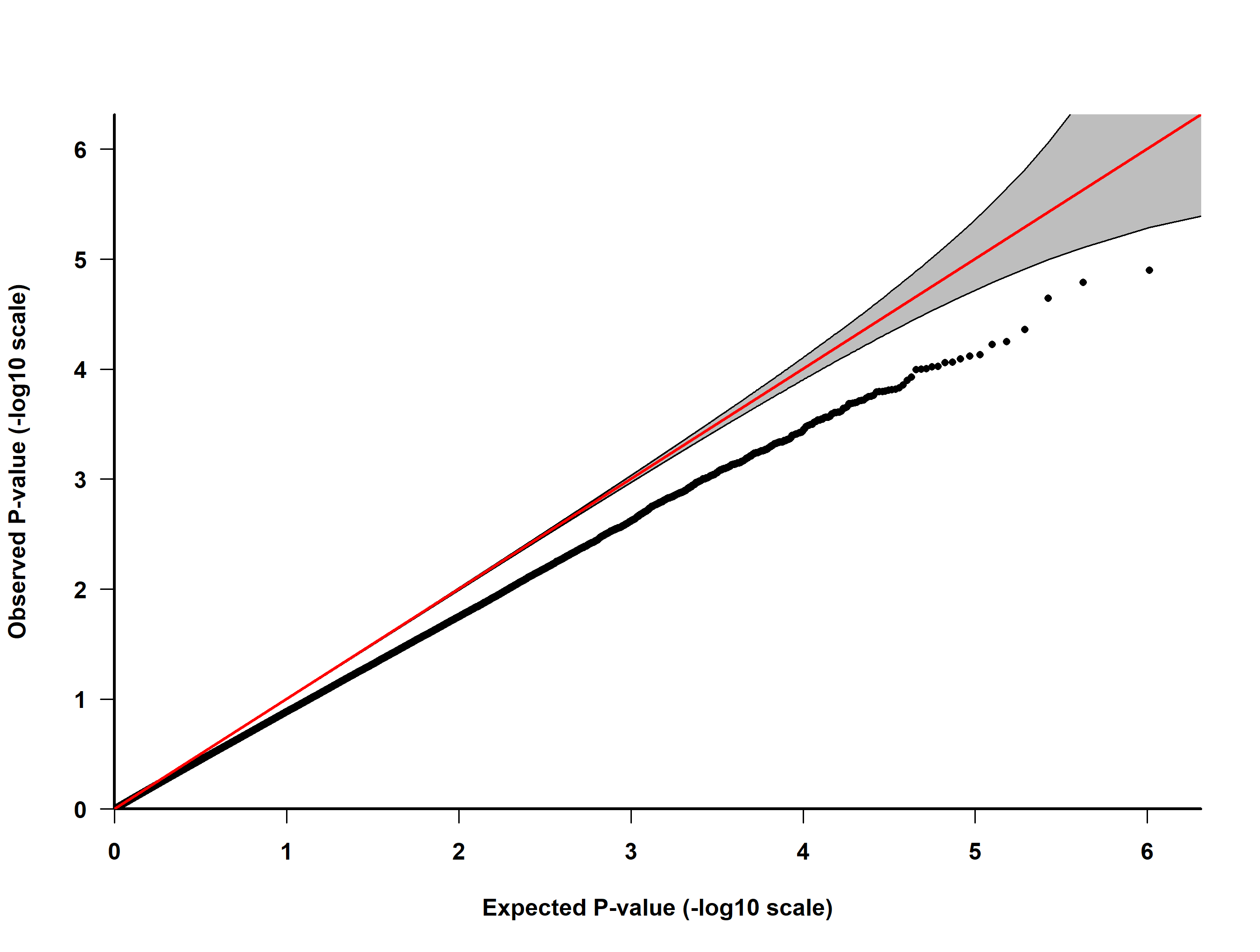


B

A

Supplementary Figure 13. QQ-plot plot of observed and expected *P*-values for urbanicity in MWAS 1 (A) and MWAS 2 (B)

The straight line is where the observed *P*-values match those expected and the shaded area is the 95% confidence interval. Genomic inflation: MWAS 1 = 0.929, MWAS 2 = 0.844


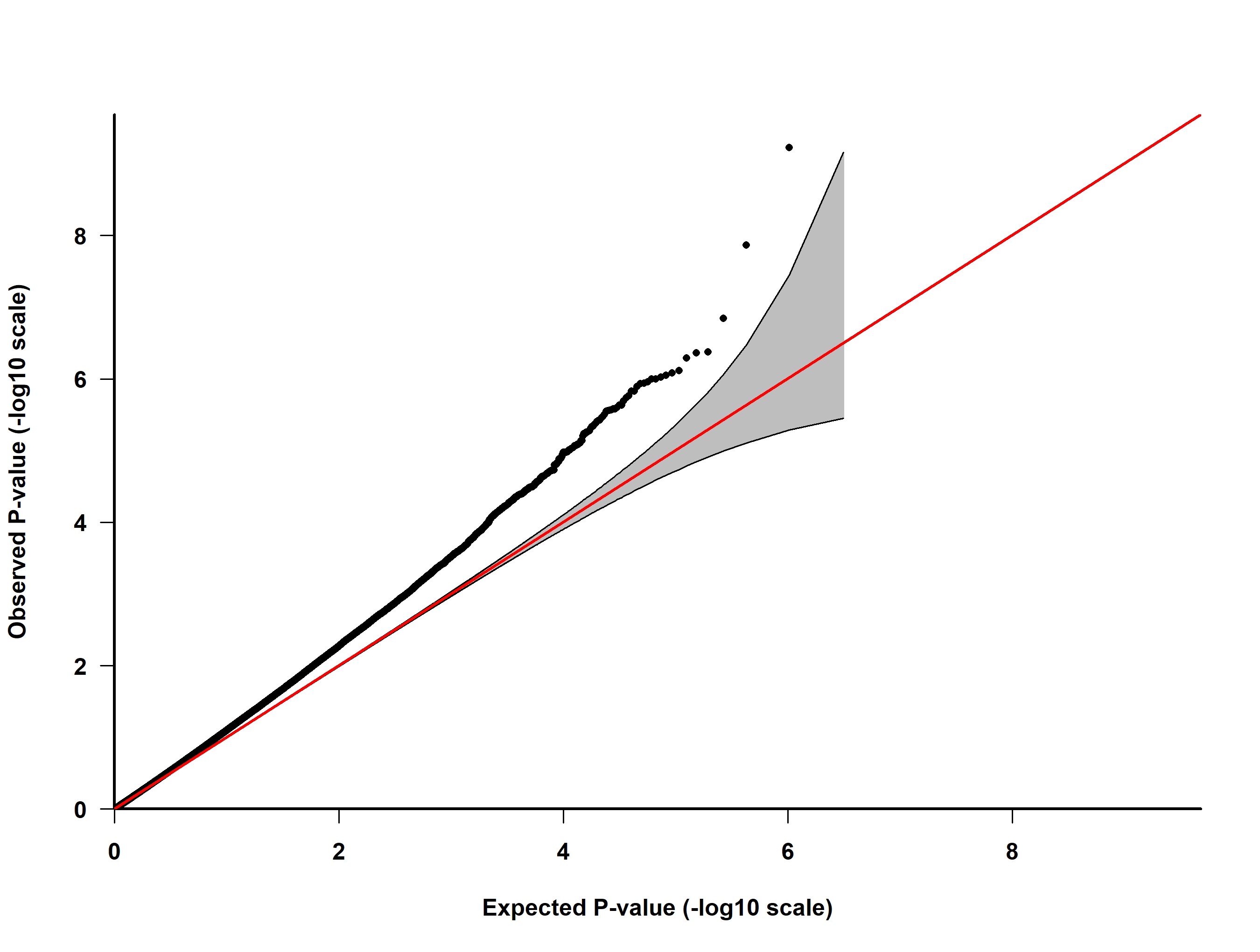

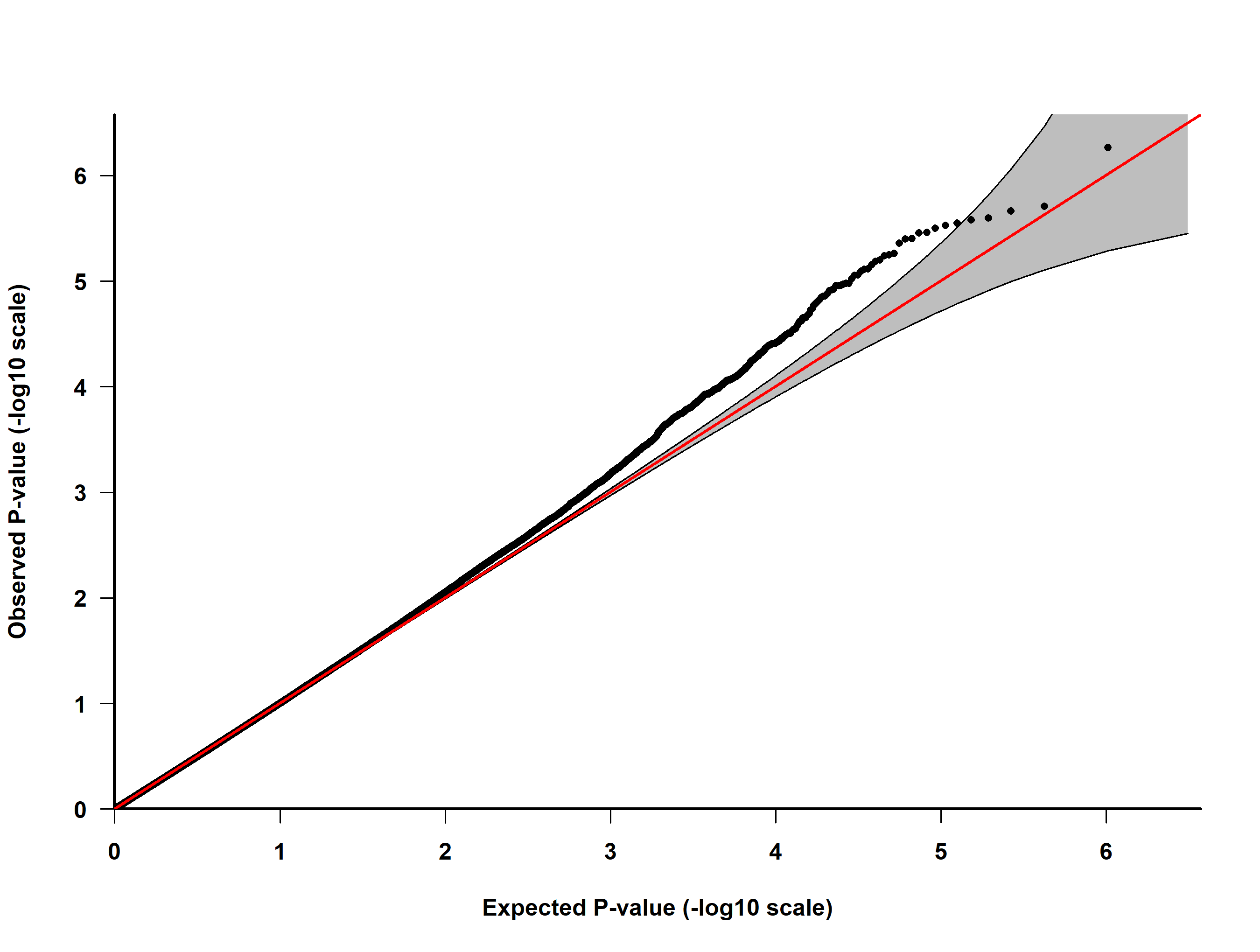


A

B

Supplementary Figure 14. QQ-plot of observed and expected *P*-values for major depressive disorder in MWAS 1 (A) and MWAS 2 (B)

The straight line is where the observed *P*-values match those expected and the shaded area is the 95% confidence interval. Genomic inflation: MWAS 1 = 1.107, MWAS 2 = 0.978


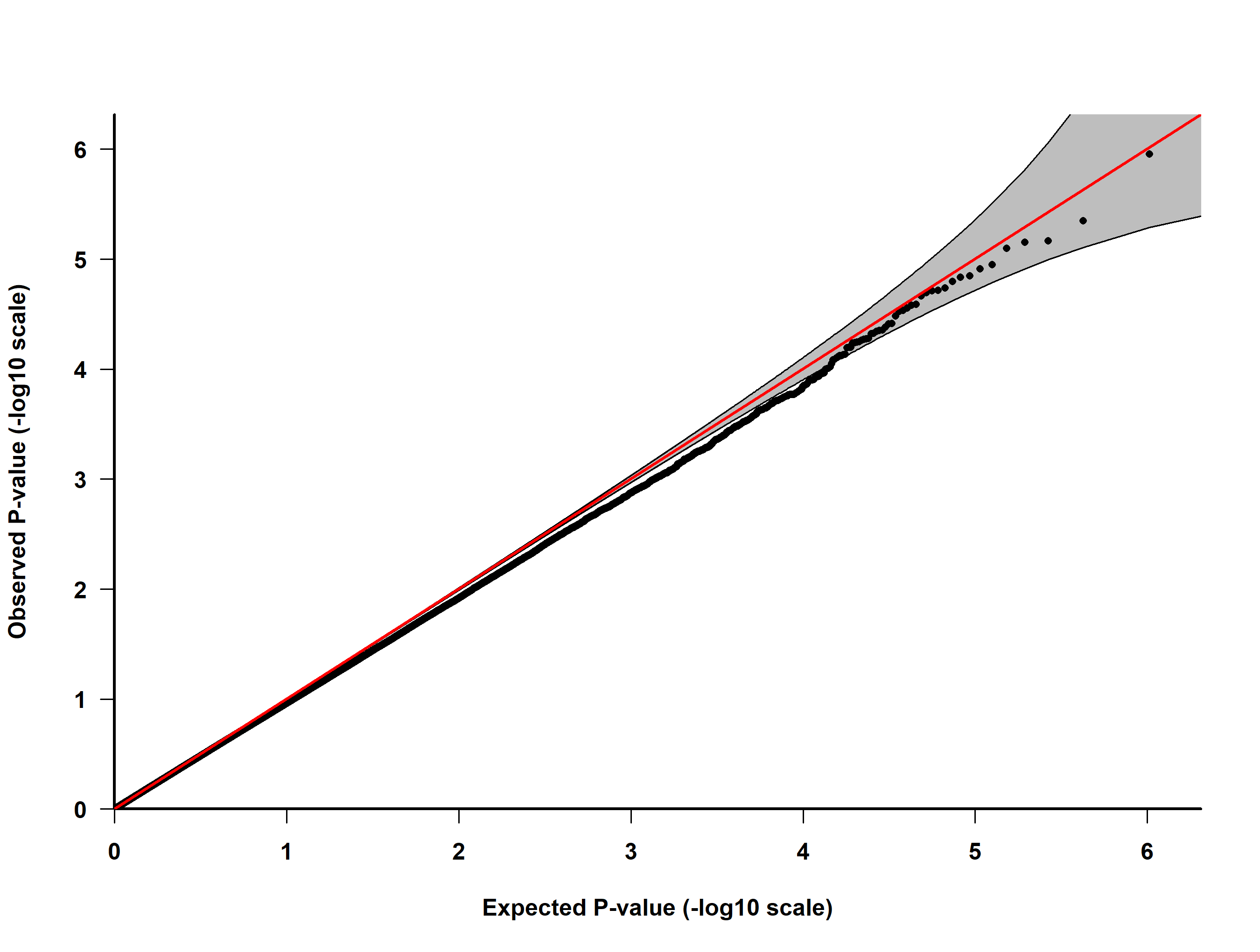

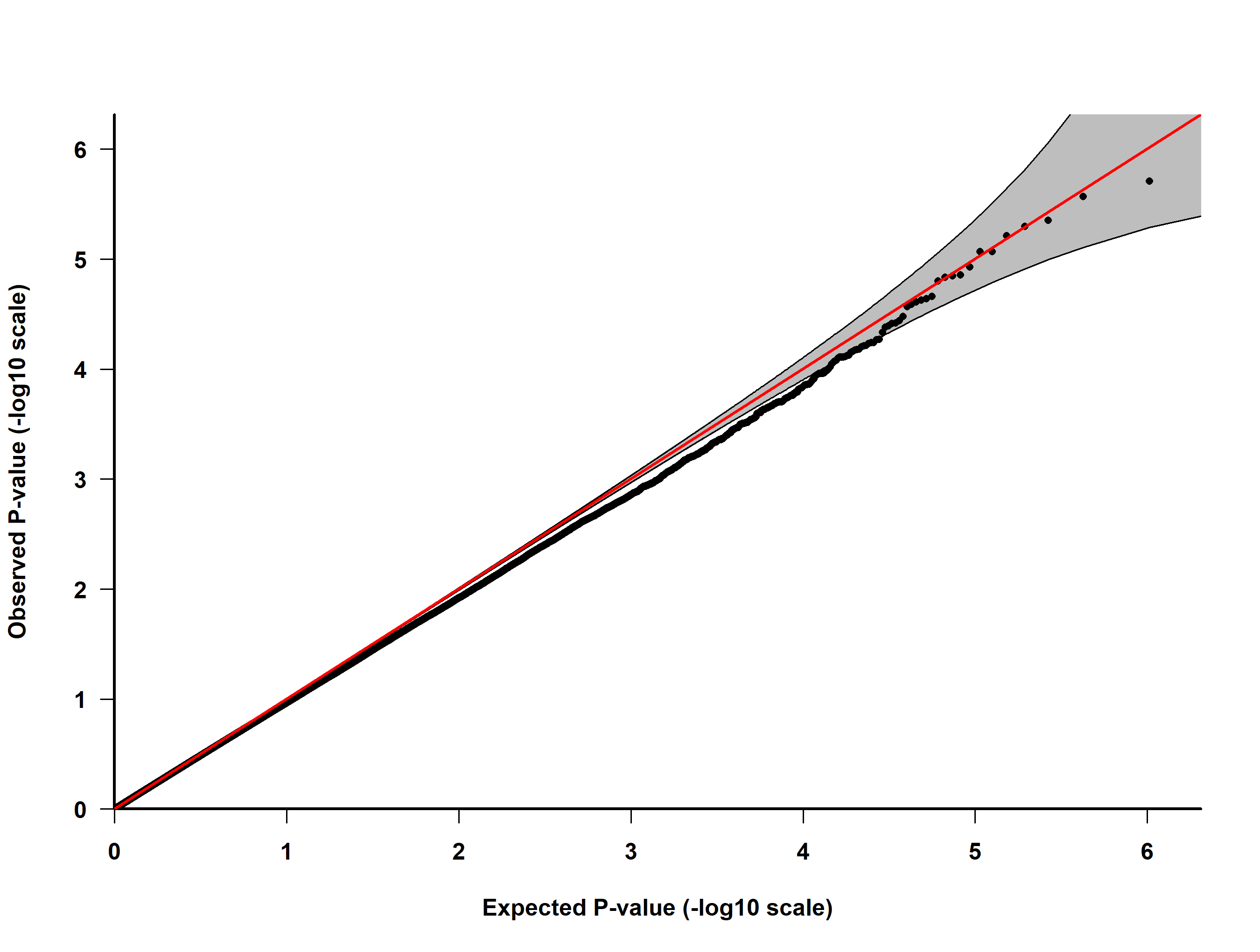


A

B

Supplementary Figure 15. QQ-plot of observed and expected *P*-values for brief resilience scale in MWAS 1 (A) and MWAS 2 (B)

The straight line is where the observed *P*-values match those expected and the shaded area is the 95% confidence interval. Genomic inflation: MWAS 1 = 0.951, MWAS 2 = 0.961


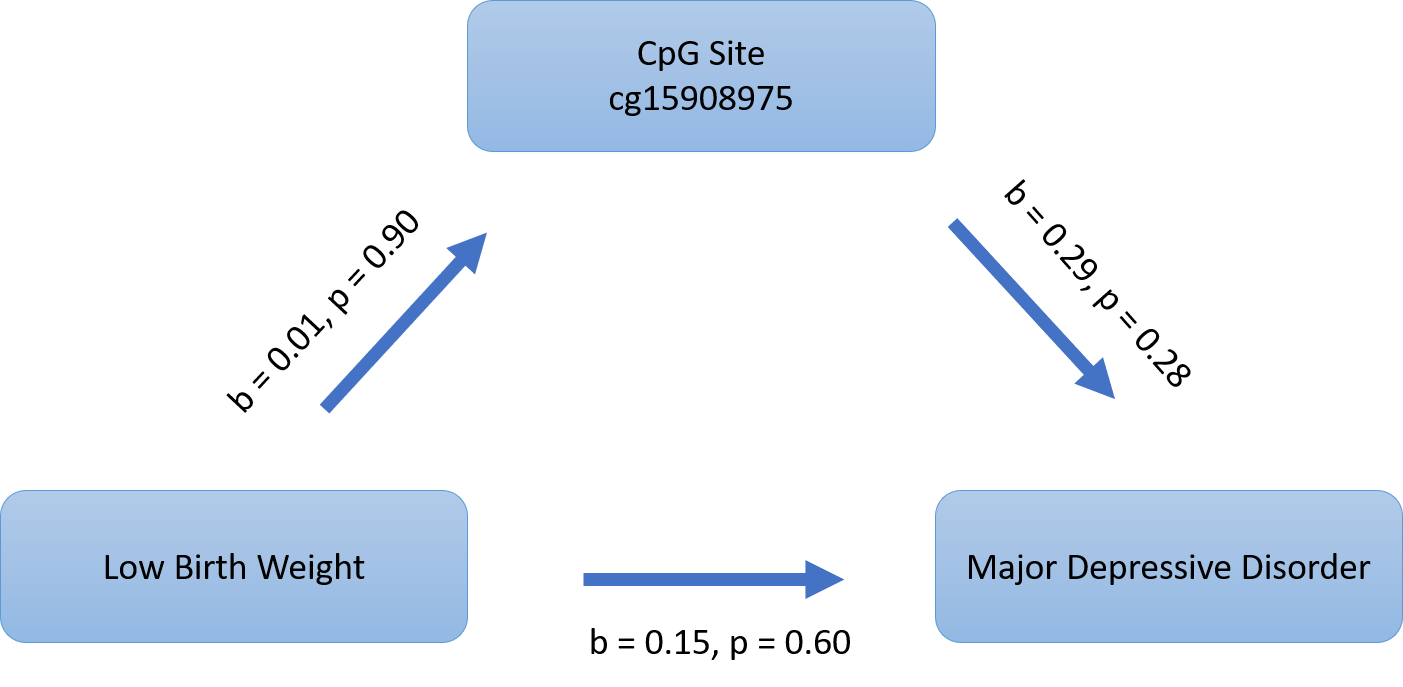


Supplementary Figure 16. Effect sizes (b) and *P*-values (p) from a mediation analysis with Low Birth Weight as the independent variable, Major Depressive Disorder as the dependent variable and cg15908975 as the mediator


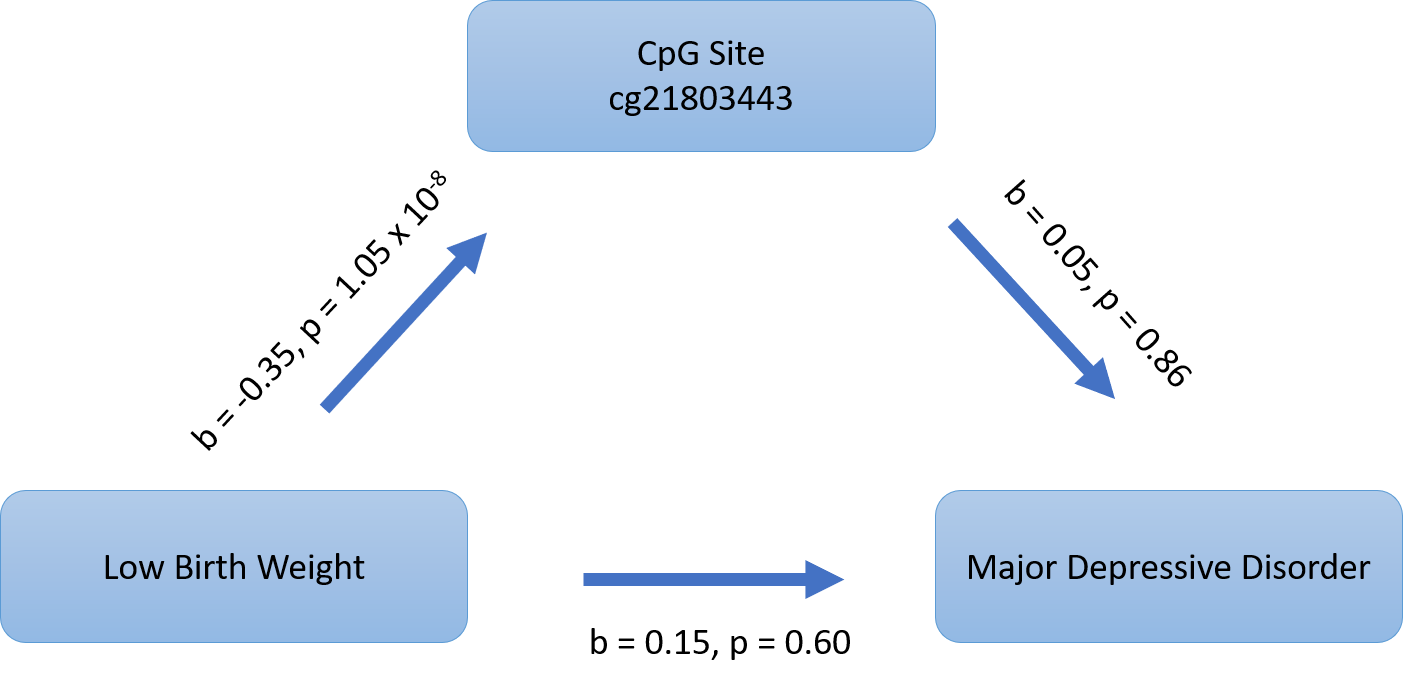


Supplementary Figure 17. Effect sizes (b) and *P*-values (p) from a mediation analysis with Low Birth Weight as the independent variable, Major Depressive Disorder as the dependent variable and cg21803443 as the mediator


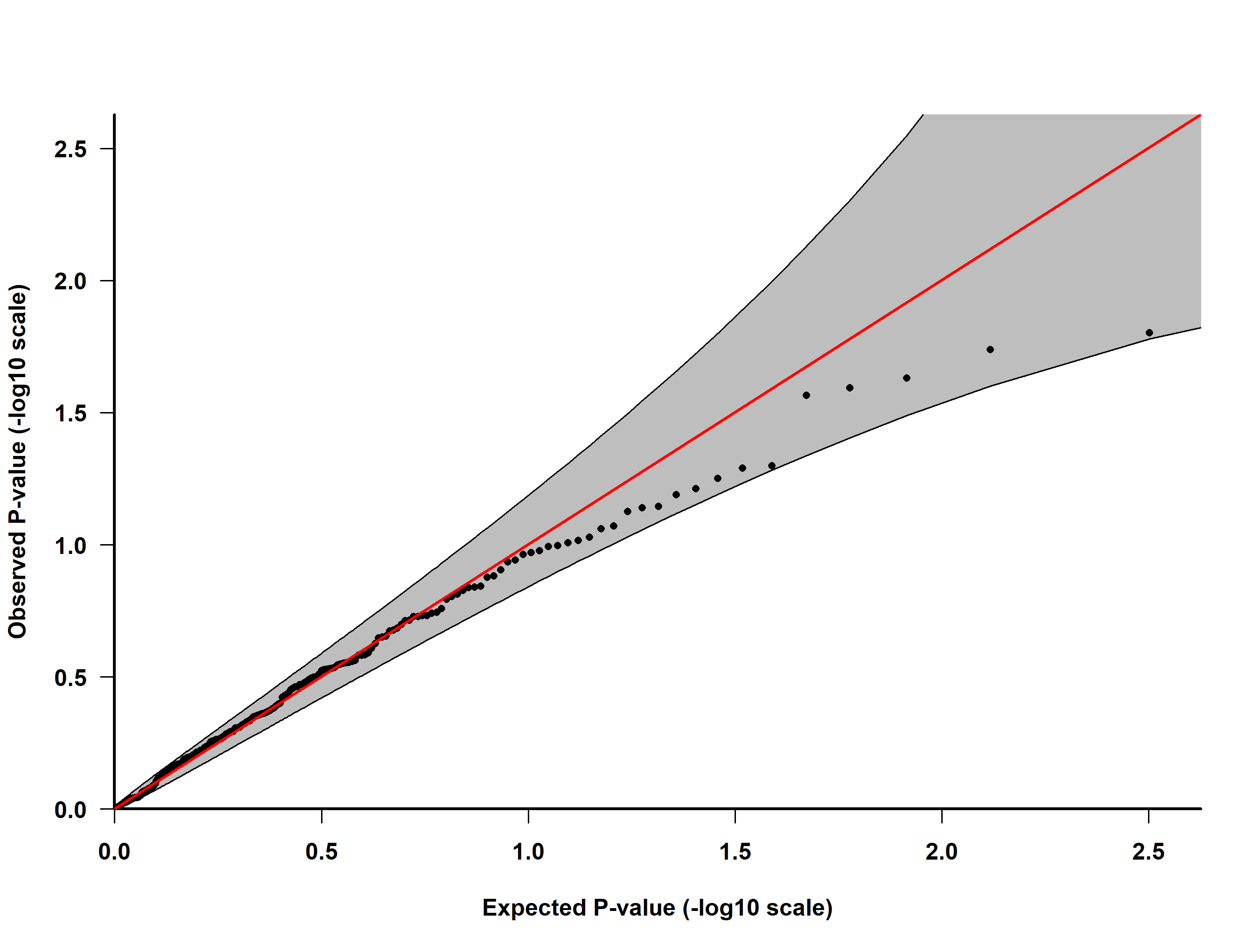


Supplementary Figure 18. QQ-plot of observed and expected *P*-values for 220 CpG sites with basophil count in the Lothian Birth Cohort

The 220 CpG sites were those associated with birth month in the Generation Scotland: Scottish Family Health Study. The straight line is where the observed *P*-values match those expected and the shaded area is the 95% confidence interval. Genomic inflation: 1.042


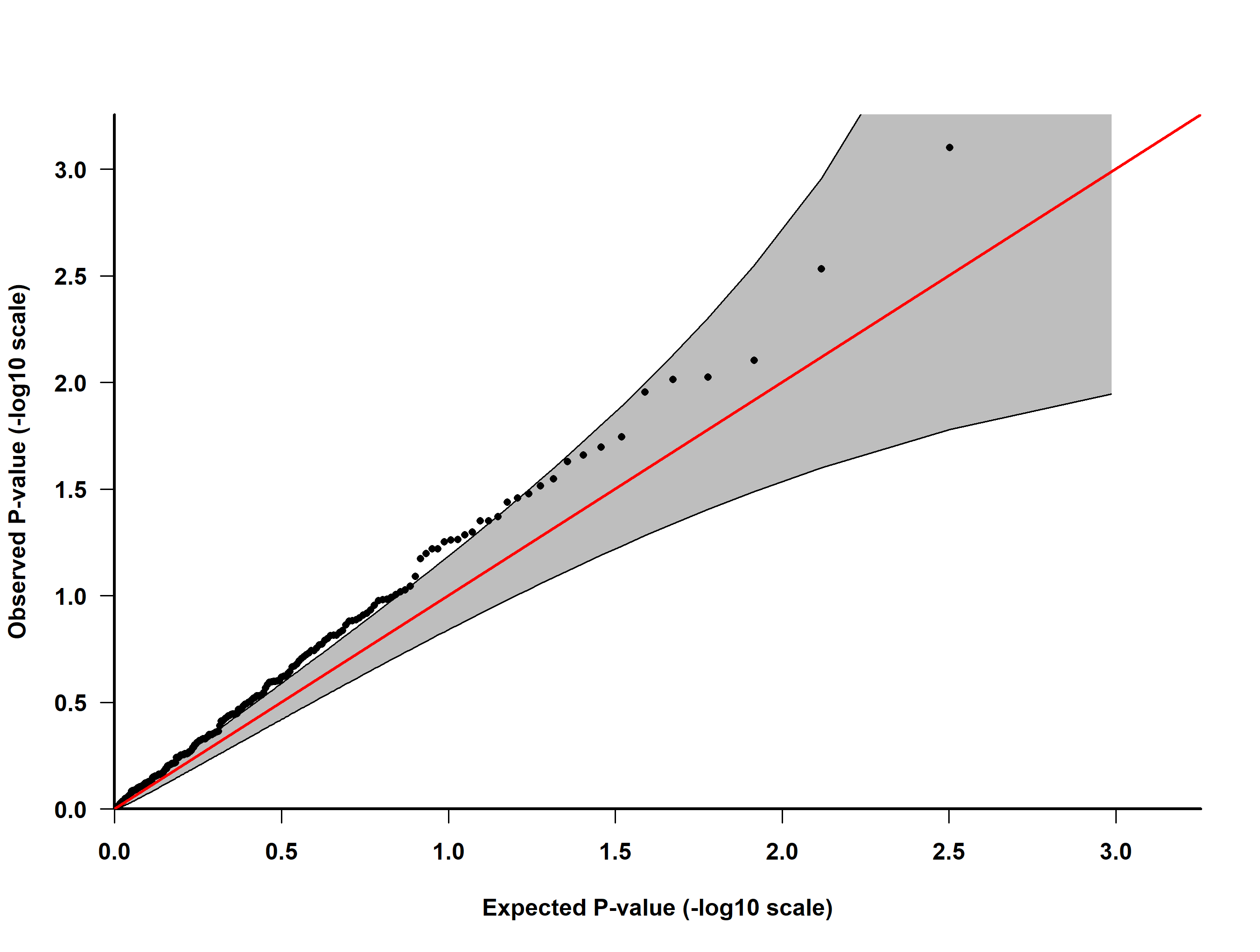


Supplementary Figure 19. QQ-plot of observed and expected *P*-values for 220 CpG sites with eosinophil count in the Lothian Birth Cohort

The 220 CpG sites were those associated with birth month in the Generation Scotland: Scottish Family Health Study. The straight line is where the observed *P*-values match those expected and the shaded area is the 95% confidence interval. Genomic inflation: 1.319


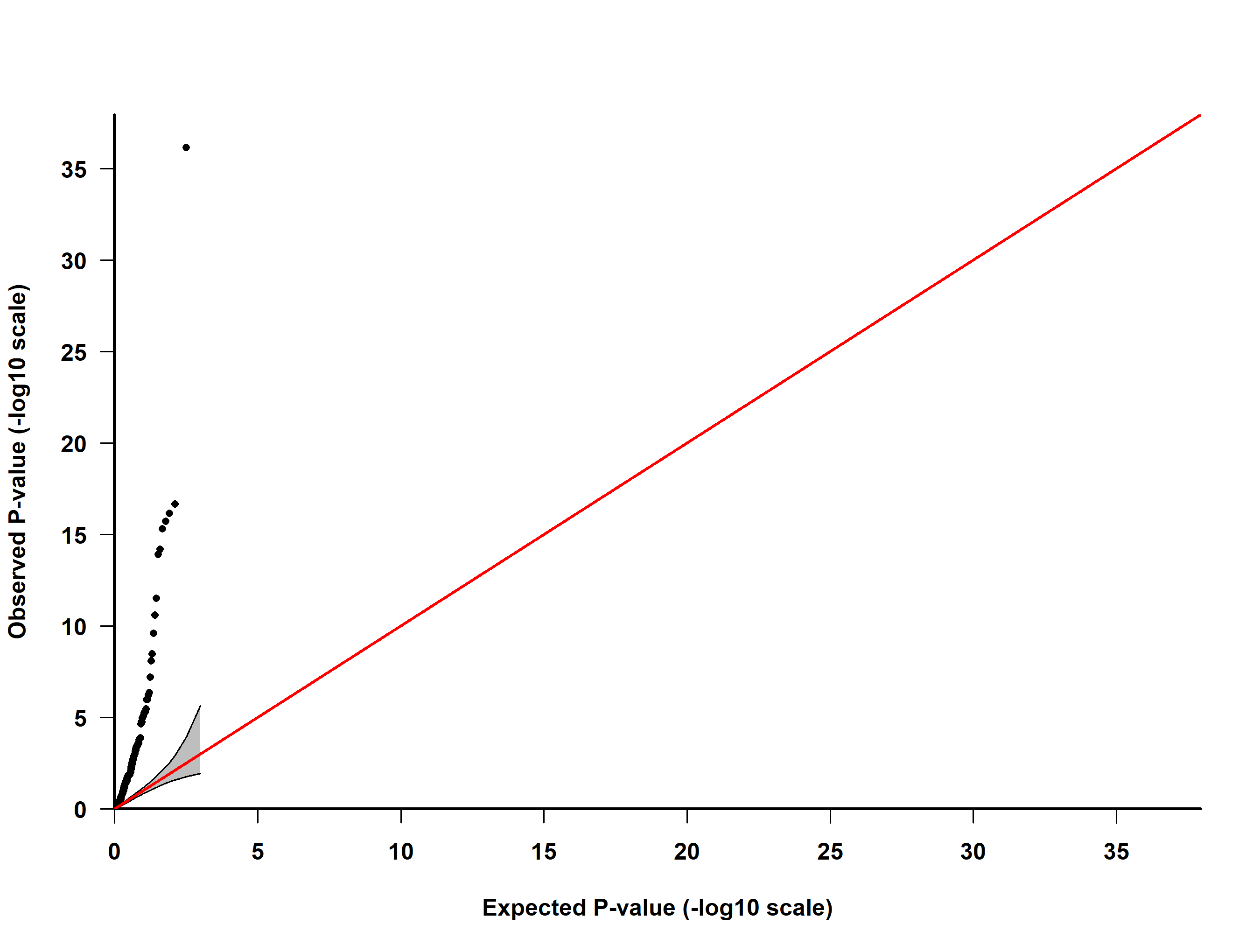


Supplementary Figure 20. QQ-plot of observed and expected *P*-values for 220 CpG sites with lymphocyte count in the Lothian Birth Cohort

The 220 CpG sites were those associated with birth month in the Generation Scotland: Scottish Family Health Study. The straight line is where the observed *P*-values match those expected and the shaded area is the 95% confidence interval. Genomic inflation: 5.841


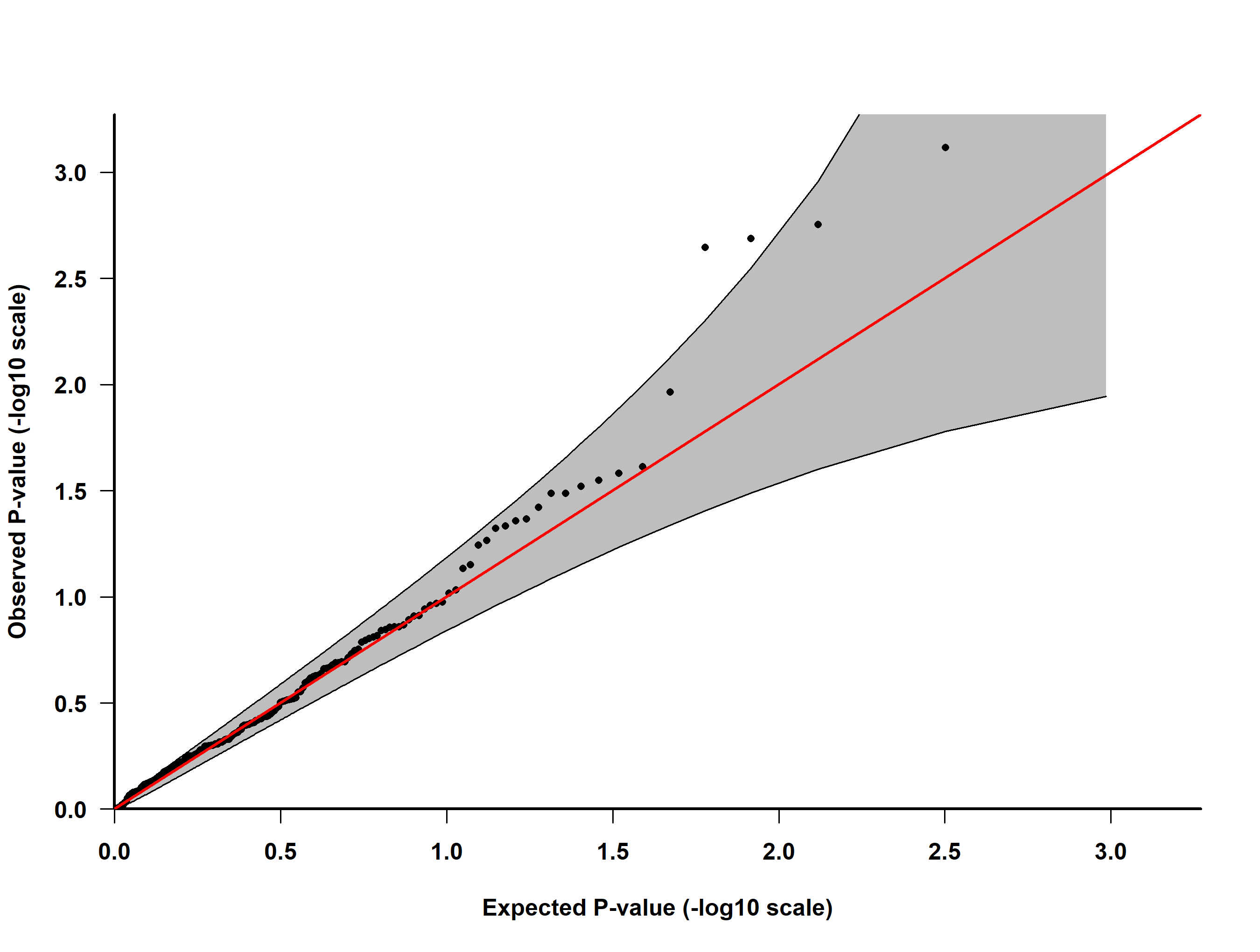


Supplementary Figure 21. QQ-plot of observed and expected *P*-values for 220 CpG sites with monocyte count in the Lothian Birth Cohort

The 220 CpG sites were those associated with birth month in the Generation Scotland: Scottish Family Health Study. The straight line is where the observed *P*-values match those expected and the shaded area is the 95% confidence interval. Genomic inflation: 1.014


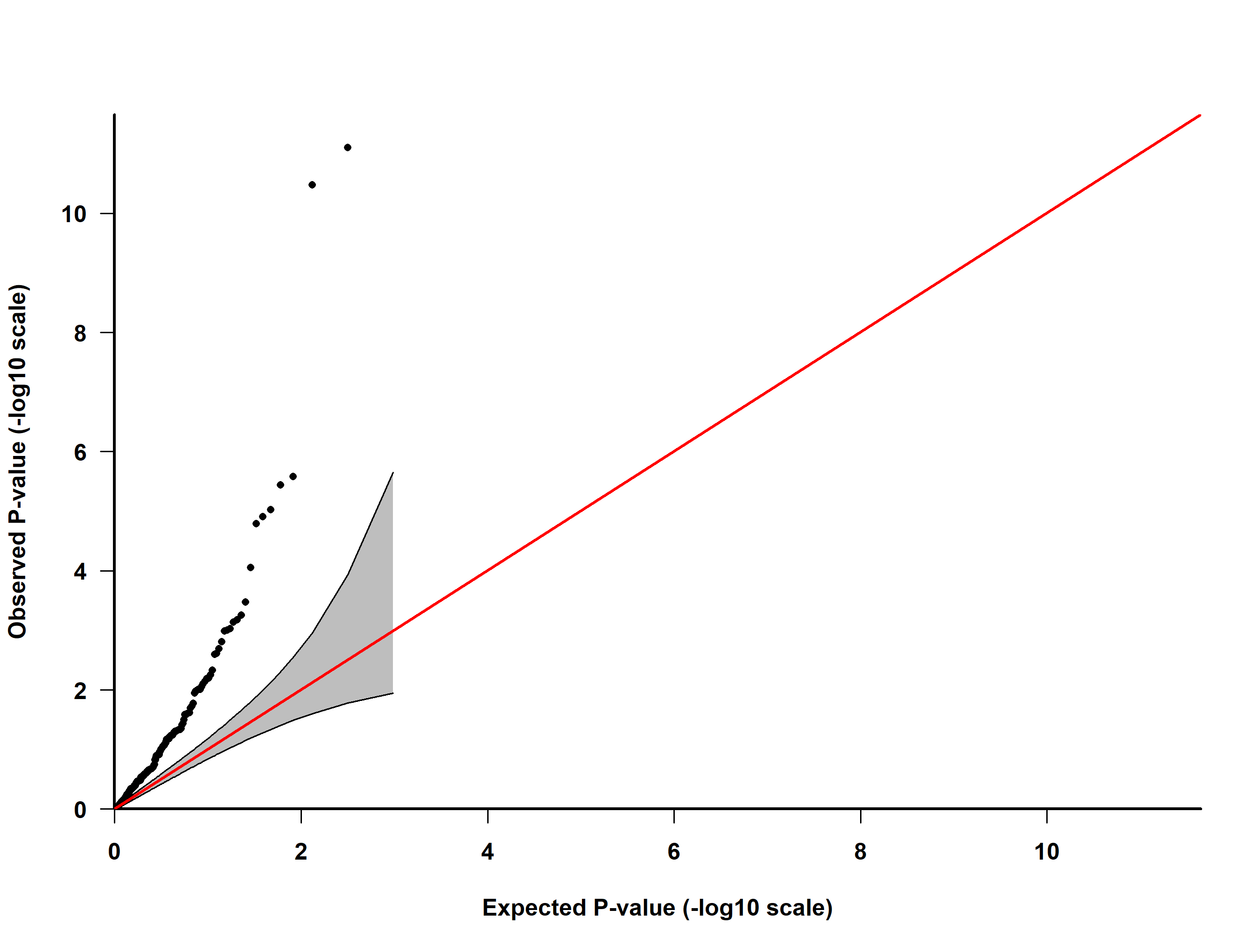


Supplementary Figure 22. QQ-plot of observed and expected *P*-values for 220 CpG sites with neutrophil count in the Lothian Birth Cohort

The 220 CpG sites were those associated with birth month in the Generation Scotland: Scottish Family Health Study. The straight line is where the observed *P*-values match those expected and the shaded area is the 95% confidence interval. Genomic inflation: 2.503
