# Supplementary material for: Methylome-wide association study of early life stressors and adult mental health
Source: Hum Mol Genet. 2021 Sep 15;31(4):651–64. doi: 10.1093/hmg/ddab274 (PMC8863421; doi:10.1093/hmg/ddab274)
Supplement: Supplementary_Information_ddab274 [file supplementary_information_ddab274.docx]

Supplementary Information

Blood Cell Type Composition

A notable difference was observed between the results from MWAS 1 and MWAS 2 for the time of year of birth phenotypes. The inclusion of predicted cell type composition as a fixed effect in MWAS 2 represents a potential source of these differences. Additional analyses were conducted to investigate blood cell type composition and are described below.

Results

In MWAS 1, there were 314 lead CpG sites (r^2^ > 0.1 within a 1 Mb window) associated with birth date (*P* < 7.01 × 10^-8^); however, in MWAS 2 there were no significant CpG sites for birth date. MWAS 2 included predicted blood cell type composition as a fixed effect, using haematological data obtained from the Lothian Birth Cohorts of 1921 and 1936 (LBC). Access to the LBC was obtained and a MWAS run on five blood cell types: basophils, eosinophils, lymphocytes, monocytes, and neutrophils. Of the 314 lead CpG sites from MWAS 1 in GS:SFHS, 220 were available in the LBC. The QQ-plots for the observed and expected *P*-values and $\lambda$ for these 220 sites in LBC are provided in Supplementary Figures 18-22 for each blood cell type. Examination of these plots revealed no inflation of *P*-values for basophil, eosinophil, and monocyte counts. However, there was noticeable inflation for neutrophil (50 out of 220 sites with *P* < 0.05; $\lambda$ = 2.503) and lymphocyte (98 out of 220 sites with *P* < 0.05; $\lambda$ = 5.841) counts. Similarly, creating methylation profile scores using the MWAS 1 effect sizes for birth date in GS:SFHS (Supplementary Table 4), revealed significant prediction of neutrophil (*P* = 7.81 × 10^-4^) and lymphocyte counts (*P* = 3.45 × 10^-7^) in the LBC. These profile scores captured 0.8% and 1.9% of the phenotypic variance of the neutrophil and lymphocyte counts, respectively. In LBC, linear regressions were conducted with each blood cell type assessed separately as the dependent variable for an association with birth date as the independent variable, although none of these regressions were significant (*P* > 0.05).

Discussion

The initial methodology of the current study was to use a single linear regression, MWAS 1. It was only after observing genomic inflation in the signal for the time of year of birth phenotypes that MWAS 2 was also included. MWAS 2 accounts for methylome-wide correlational structure and makes additional adjustment for predicted cell type composition. The non-significance of any of the associated MWAS 1 variants in MWAS 2, suggests cell type composition as the likely cause of the inflation, which was confirmed by our analysis in the LBC. Therefore, correcting M-values for cell type composition using the Houseman algorithm (1) may not be adequate, as there remained a detectable effect of cell type composition in the GS:SFHS data. Furthermore, if we had only run MWAS 2 we would not have observed the effect of time of year on birth on cell type composition. In future, it may be preferable to run multiple regression models on DNA methylation data.

Materials & Methods

To investigate cell type composition haematological and DNA methylation data from 1,308 participants of the LBC was obtained (2, 3), mirroring the development of the OSCA package used in MWAS 2. A similar protocol to that described by Zhang, et al. (4) was used to identify effect sizes and *P*-values between 214,923 CpG sites and the cell counts of basophils, eosinophils, lymphocytes, monocytes, and neutrophils.

First, the significant lead CpG sites (*P* < 7.01 × 10^-8^) identified for birth date for MWAS 1 in GS:SFHS were examined in the LBC for an association with the five cell type compositions using QQ-plots and $\lambda$. Lead sites were identified by retaining the site with the lowest *P*-value amongst sites with an r^2^ greater than 0.1 within a 1 Mb window. Second, methylation profile scores were created for individuals in the LBC using the effect sizes from MWAS 1 for birth date in GS:SFHS. These profile scores were created using lead CpG sites at seven *P*-values thresholds (< 10^-7^, < 10^-6^, < 10^-5^, < 10^-4^, < 10^-3^, < 10^-2^, < 10^-1^). A simple linear regression was then used to calculate the association between the profile scores for birth date and the five measured cell type compositions, after adjusting for the sex and age of participants as fixed effects. A Bonferroni correction based on the five of cell types analysed required a *P*-value < 0.01 (α = 0.05 / 5) for an association.

The LBC cohort was also used to conduct linear regressions with basophil, eosinophil, lymphocyte, monocyte, and neutrophil cell counts fitted in turn as a dependent variable and birth date as the independent variable.

References

1 Houseman, E.A., Accomando, W.P., Koestler, D.C., Christensen, B.C., Marsit, C.J., Nelson, H.H., Wiencke, J.K. and Kelsey, K.T. (2012) DNA methylation arrays as surrogate measures of cell mixture distribution. *BMC Bioinformatics*, **13**, 86.

2 Taylor, A.M., Pattie, A. and Deary, I.J. (2018) Cohort profile update: the Lothian Birth Cohorts of 1921 and 1936. *International Journal of Epidemiology*, **47**, 1042-1042r.

3 Starr, J.M. and Deary, I.J. (2011) Sex differences in blood cell counts in the Lothian Birth Cohort 1921 between 79 and 87 years. *Maturitas*, **69**, 373-376.

4 Zhang, F., Chen, W., Zhu, Z., Zhang, Q., Nabais, M.F., Qi, T., Deary, I.J., Wray, N.R., Visscher, P.M., McRae, A.F. *et al.* (2019) OSCA: a tool for omic-data-based complex trait analysis. *Genome Biology*, **20**, 107.
